# Health Effects of Living Near Petroleum and Biofuel Refineries: A Systematic Evidence Map and Scoping Review

**DOI:** 10.1007/s40572-026-00530-0

**Published:** 2026-03-14

**Authors:** Marinelle Villanueva, Qi Meng, Jenni A. Shearston, Rachel Morello-Frosch, Lara J. Cushing

**Affiliations:** 1 Department of Environmental Health Sciences, Fielding School of Public Health, University of California, Los Angeles, Los Angeles, CA USA; 2 Department of Integrative Physiology, University of Colorado Boulder, Boulder, CO USA; 3 Department of Environmental Science, Policy, and Management, School of Public Health, University of California, Berkeley, Berkeley, CA USA

## Abstract

**Purpose of Review:**

Petroleum refineries are significant sources of toxic contaminant emissions globally that threaten the health of refinery workers and nearby populations. We conducted a scoping review of the epidemiological evidence concerning acute and chronic health impacts of petroleum and biofuel refineries on surrounding communities. We evaluated the evidence using risk-of-bias assessments and catalogued findings into an evidence map to identify gaps in the literature. We systematically searched studies published by August 2024 on PubMed, Embase, and Web of Science.

**Recent Findings:**

Our review includes 54 studies published between 1977 and 2024 in 15 international regions. Most studies focused on cancer, respiratory effects, biomarkers of effect, acute symptoms, and cardiovascular effects; fewer studies examined other chronic diseases, reproductive or birth outcomes. Multiple studies find evidence that residential proximity to refineries is associated with higher risks of leukemia, asthma, bronchitis, respiratory symptoms, and healthcare utilization. 13 studies found evidence of dose-response relationships between proximity or duration of residence near a refinery and severity of risk. Common study limitations included imprecise exposure measures, limited control for confounding, and cross-sectional designs. Recent developments have improved causal inferences, including air pollutant dispersion modeling, quasi-experimental designs, and individual-level exposure measures.

**Summary:**

The literature suggests a range of acute and chronic health effects among adults and children living near refineries. Additional longitudinal studies and studies of biorefineries are needed, especially as the energy transition from petroleum to bio-based transportation fuels accelerates. Our review underscores the need for additional measures to protect the health of refinery communities.

**Supplementary Information:**

The online version contains supplementary material available at 10.1007/s40572-026-00530-0.

##  Introduction

 Petroleum refining, a key component of the global energy and transportation fuel supply, is a leading source of hazardous pollution. Refineries process over 100 million barrels per day of petroleum (crude oil) into liquid fuels in 98 countries, with production capacity doubling over the last 50 years [1, 2]. Globally, the petroleum refining sector is the largest source of toxic emissions in the oil industry and is the third largest stationary source of greenhouse gas emissions, accounting for 34 gigatons of carbon dioxide from 2000-2021 [3, 4]. In the United States (US) – the world’s largest producer and consumer of petroleum – an estimated 6.1 million people live within 3 miles (5km) of a petroleum refinery [1, 5]. The rapid growth of this industry poses significant threats to the health, environment, and safety of nearby populations.

 Biofuels have emerged as a potential alternative to petroleum-based transportation fuels that can reduce greenhouse gas emissions and mitigate climate change. Several petroleum refineries have already transitioned to processing bio-based feedstocks, such as biomass, instead of crude oil [6]. Global production of biofuels has increased six-fold since 2000 and is projected to contribute up to 27% of global transportation fuel demand by 2050 [7]. However, biofuel refineries also emit toxic air pollutants such as particulate matter, volatile organic compounds, sulfur dioxide, and nitrogen dioxide [8–10]. The extent to which they may result in reduced pollutant emissions relative to petroleum refineries is poorly understood. As biorefineries become an increasingly important share of the energy economy, it is necessary to evaluate the public health implications that this transition will have on local communities.

 The health burden of refinery emissions is disproportionately borne by nearby communities. Households in poverty, Hispanics and African Americans are more likely to live near US refineries, which tend to be located in urban centers near highways and other industrial facilities where they contribute to cumulative air pollution burdens [11]. A recent meta-analysis focused on cancer among workers and residents living near petrochemical industry and oil extraction sites found evidence of higher risk of childhood leukemia [12]. Prior reviews have documented health impacts among workers and nearby residents of petrochemical and oil extraction complexes including cancer and acute respiratory, immunological, reproductive, and neurological effects [13–16]. However, to our knowledge, no review to date has assessed the broad range of health outcomes associated specifically with refineries in local communities.

 To address this gap, we developed a systematic evidence map and scoping review to critically evaluate the extent of published literature and future research needs on the potential acute and chronic health risks associated with residence near petroleum and biofuel refineries. Our study expands on prior reviews by focusing on residential communities as opposed to workers and catalogues the breadth of health impacts, including cancer, respiratory outcomes, cardiovascular outcomes, acute symptoms, biomarkers of effect, birth outcomes, healthcare utilization, and mortality. We included studies that evaluated health impacts based on short- and long-term residential proximity or exposure to refinery emissions, as well as facility closures, flares, and fires. Further, we assessed whether health risks vary by distance of residence from refinery locations. We additionally include biofuel refineries in order to inform future research on the health implications of greenhouse gas mitigation efforts to decarbonize the transportation fuel supply. Our systematic evidence map can be useful for decision-makers, community members, and researchers by facilitating access to existing evidence.

## Methods

### Search Strategy

 We conducted a scoping review to evaluate the existing epidemiological literature on the community health implications of petroleum and biofuel refineries that produce liquid transportation fuels. This review was conducted in accordance with the reporting guidelines for systematic evidence maps and the Preferred Reporting Items for Systematic reviews and Meta-Analyses extension for Scoping Reviews (PRISMA-ScR) [17, 18]. In consultation with a university librarian, we employed a broad search strategy with keywords and MeSH terms related to refineries, human health outcomes, and residential proximity. Studies were identified in PubMed, Embase, and Web of Science from database searches initiated in February 2024 with forward and backward searches through August 2024. A detailed description of the search strategy is presented in Supplemental Table 1.

### Eligibility Criteria

We developed a PECO framework (Population, Exposure, Comparators, and Outcomes) to define our eligibility criteria:

- Population: Any population residing near petroleum or biofuel refineries, regardless of sex, age, or geographical region.
- Exposure: Proximal to or exposed to pollutants from refineries.
- Comparators: Reference or comparison populations not or less proximal to refineries; exposed to lower or no levels of pollutants from refineries; or the same population serving as their own control when comparing outcomes over time or before-and-after an event of interest.
- Outcomes: Cancer, mortality, non-cancer morbidity (including respiratory effects, cardiovascular effects, acute symptoms, birth outcomes, and healthcare utilization), and biomarkers of effect.

Studies published in any language from the earliest time available to the present were eligible for inclusion. We excluded animal toxicology studies, review articles, health risk assessments, biomonitoring studies that did not include biomarkers of effect, occupational health studies, and populations co-exposed to other unrelated industries. Additionally, we included observational studies that quantified associations with human health outcomes, omitting purely descriptive studies. Studies pertaining to petrochemical industrial complexes were included if a petroleum refinery was specifically assessed, given that refineries are often co-located within a larger industrial complex. Studies evaluating exposure to multiple industries were only retained if associations attributable to refineries were measured.

### Study Selection

 After removing duplicate records, two independent reviewers (MV and QM) screened the titles and abstracts using our pre-defined PECO eligibility criteria. Records selected during screening were considered eligible for full-text review if the title and/or abstract contained keywords relevant to human health risks near a refinery. We performed forward and backward citation searches for all eligible records, risk assessments, and review articles to capture any other relevant citations that may have been missed during the database searches. Any discrepancies during the screening phase were resolved by discussion between five reviewers to reach a consensus (MV, QM, JAS, RMF, and LJC).

### Data Extraction 

 We (MV, QM, and JAS) reviewed the full texts of all eligible manuscripts. For each eligible manuscript, two independent reviewers were randomly assigned to extract data. The following information from each study was extracted into a spreadsheet: study location, time period, design, sample size, statistical analysis, exposure/s, data source/s, outcome/s, confounder/s, effect estimate/s, confidence interval/s, and effect measure modification. The reviewers decided whether to include a study in the final synthesis based on the eligibility criteria, while blinded to the other reviewer’s decision. Disagreements between reviewers regarding study inclusion were settled via discussion among five reviewers (MV, QM, JAS, RMF, and LJC).

### Critical Appraisal of Individual Sources of Evidence 

 All studies selected for full-text review were critically appraised using a thirteen (13) item checklist to assess sources of bias (Supplemental Table 2). These criteria were adapted from critical appraisal tools for observational studies [19]. We utilized existing criteria for each study design (cohort, case-control, cross-sectional, ecological) to develop a standardized tool to assess study quality across various designs for the following risk-of-bias domains: selection strategy, exposure assessment, study design, outcome assessment, selective outcome reporting, confounding, statistical analysis, and conflict of interest. Each reviewer independently classified all evaluation questions as yes-or-no for each study and documented justification for each criterion, while blinded to the other reviewer’s assessment (MV, QM, and JAS).

 We calculated the mean domain-specific risk-of-bias score between two reviewers for each study. Yes-or-no scores were converted to 1 and 0. For domains with two criteria, scores were normalized to a 0-to-1 scale. To avoid assigning equal or arbitrary weights to domains, we did not derive overall critical appraisal scores to preserve the distinct contribution of risk-of-bias domains to observational study quality [20]. By reporting domain-specific ratings, we sought to elucidate the sources of bias in the studies included. Domain-specific ratings were categorized as high, moderate, or low risk of bias if the mean score was less than or equal to 0.50, between 0.51 and 0.99, or 1.0, respectively. These ratings were visualized as heat maps for each study within each health outcome category.

### Synthesis of Results

 Health outcomes were organized into six categories: cancer, respiratory effects, acute symptoms, biomarkers of effect, cardiovascular effects, and other adverse outcomes. We categorized study characteristics for the type of refinery, region, exposure methods, study design, population, and distance between residence and refinery locations. Extracted data and critical appraisal scores from each reviewer were combined into a harmonized spreadsheet and minor discrepancies in the data extracted and critical appraisal assessments between the two reviewers were flagged and revised after secondary review (MV, JAS, QM).

 We reported the direction of observed effects (adverse or not adverse) when comparing the most exposed to the reference group. A finding was classified as *adverse* if refinery-related emissions or proximity were linked to adverse health effects in the most exposed vs. reference group – for instance, if higher pollutant concentrations, closer proximity to a refinery, longer duration of residence, or pre-closure periods were associated with increased risk. Conversely, an association was classified as *not adverse* if findings suggested reduced risk.

 Studies often tested associations for multiple health outcomes, exposure assessment methods, and/or population stratum. To capture the full scope of outcomes, we used individual effect estimates and statistical tests (hereafter referred to as *findings*) as the unit of analysis when summarizing results. We define one finding as a single estimated association between one exposure and one outcome in one stratum. One study could thus contribute more than one finding. For example, a study examining two exposure methods, such as two categories of distance from a refinery, and two health outcomes contributed four findings. Associations stratified by sex or age were counted as separate findings only when pooled estimates were not available. We report both statistically significant and non-significant findings to provide a comprehensive representation of the evidence and minimize selective reporting that may introduce bias [21, 22]. Statistical significance was determined at α = 0.05. 

 We catalogued the evidence in a systematic evidence map to present the study characteristics for the literature included in this review. Systematic evidence maps (SEMs) offer a novel, evidence-based tool to characterize the extent of available evidence and to identify areas of consistency and scientific gaps [23]. One author (MV) developed a systematic evidence map as an interactive web-tool on Tableau Public, providing a queryable summary of study characteristics that can be updated as the evidence base grows. A bar graph in the systematic evidence map visualizes the number of studies reporting adverse or mixed results for a given health outcome category, with mixed results indicating a study reported both adverse and null or not adverse findings. The number of studies pertaining to each study characteristic are visualized as heat maps to represent the distribution of studies, with darker shades indicating a greater number of studies. A world map shows the number of studies conducted in each geographic region. Users can interact with the map by filtering data according to study characteristics of interest, for instance, petroleum or biofuel refineries, health outcome category, region, or exposure assessment methods. Our systematic evidence map is accessible online at the following link: https://public.tableau.com/app/profile/refineryreview/viz/Refinery-SEM/EvidenceMap.

 We capture the certainty of evidence through a comprehensive synthesis of findings with complementary presentation of summary tables and graphs in this text. We describe the number and proportion of adverse and not adverse findings and visualize the overall number of adverse, not adverse, and null findings for each health outcome category as bar graphs. To minimize reporting unstable associations arising from small stratum sizes and multiple comparisons, we report only findings with at least 25 cases per stratum [24, 25]. For example, strata are defined by cancer type, exposure group, and sex in cancer studies only reporting sex-specific findings. 

 Throughout the text, we use the terms *petroleum refineries* and *refineries* to refer to oil refineries, which are often described interchangeably in the literature. Biofuel refineries are explicitly identified where relevant. We use the term *relative risk* to summarize findings in the text and the exact measure of association estimated in each study is detailed in the summary tables. 

 We utilized Zotero v6.0.36, Microsoft Excel, ArcGIS Pro v3.2.0, R programming v4.3.2, and Tableau Public for citation management, data extraction, analysis, visualization, and systematic evidence mapping.

## Results

### Search Results

**Fig. 1:**
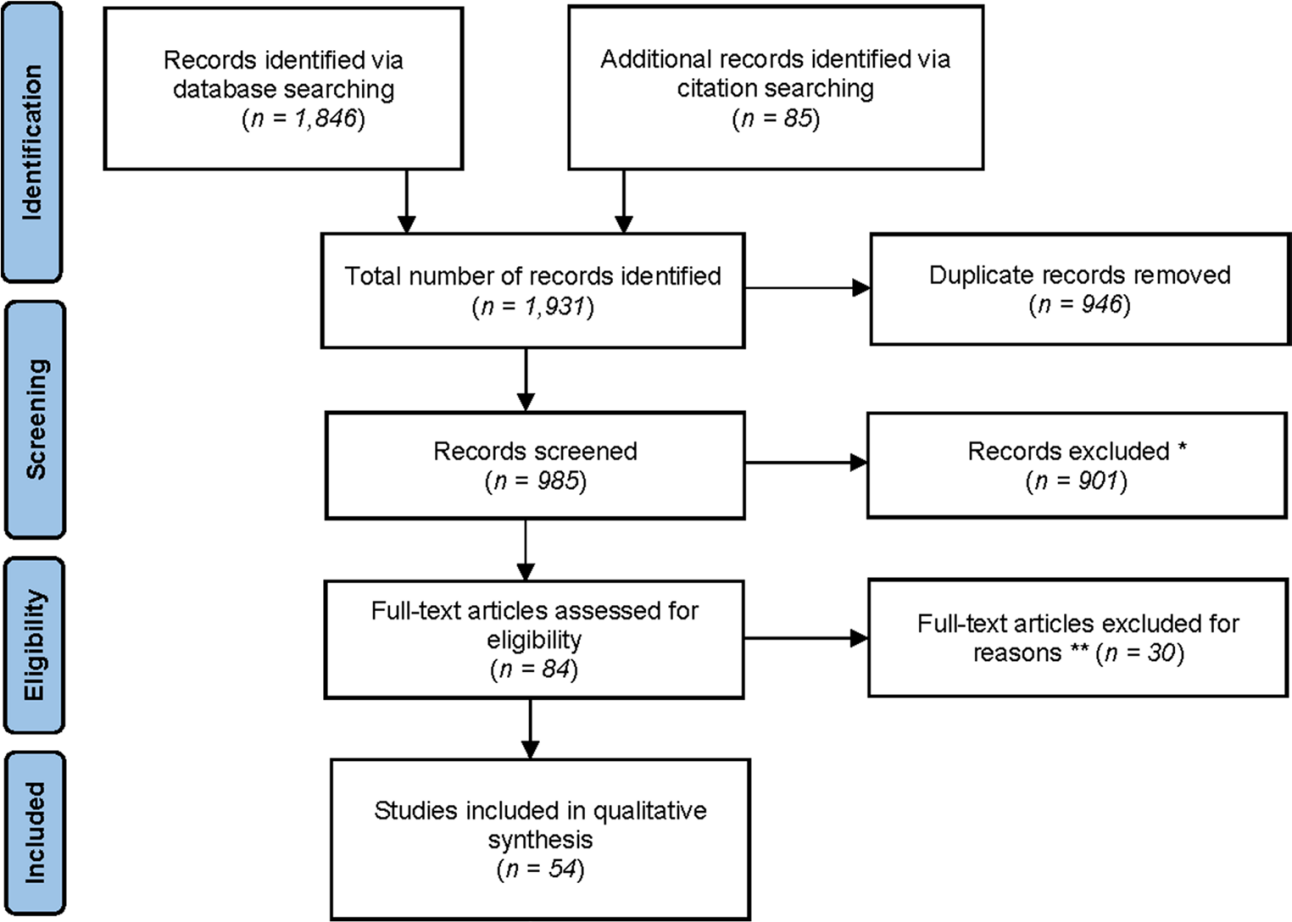
PRISMA flow diagram for identification, screening, and selection of studies. 54 studies are included in this review * Studies that did not meet eligibility criteria based on the title and abstract were excluded ** Full-text articles not meeting the eligibility criteria were excluded for the following reasons: health risk assessments, occupational health studies, containing only qualitative data, exposure of interest was unrelated, multiple unrelated industries were co-occurring where effect estimates specifically attributable to refineries were not available, and full-text articles were not available

 A total of 1,931 records were initially identified, and 946 duplicates were excluded. Of the 985 titles and abstracts that we screened, 84 manuscripts were eligible. We reviewed 84 full-text manuscripts, and 54 studies met our inclusion criteria for the final synthesis (Fig.1). Excluded studies typically consisted of health risk assessments, occupational health studies, did not contain quantitative associations, or estimates attributable to refineries were not available for populations co-exposed to unrelated industries (e.g. coal, power plant, or metal).

### Evidence Map

 We catalogued the literature into a systematic evidence map for community health effects near petroleum and biofuel refineries into categories of health outcomes, type of refinery, region, exposure methods, and study designs. The presented tables and graphs identified areas of consistency, scientific gaps, and research needs. Few studies evaluated the same exposure-outcome combinations using comparable exposure measures, such as specific air pollutants or distance-based metrics to refineries. Our systematic evidence map is freely accessible online as an interactive and user-friendly dashboard where users can query and download data for the existing studies to date: https://public.tableau.com/app/profile/refineryreview/viz/Refinery-SEM/EvidenceMap.

### General Findings

**Fig. 2:**
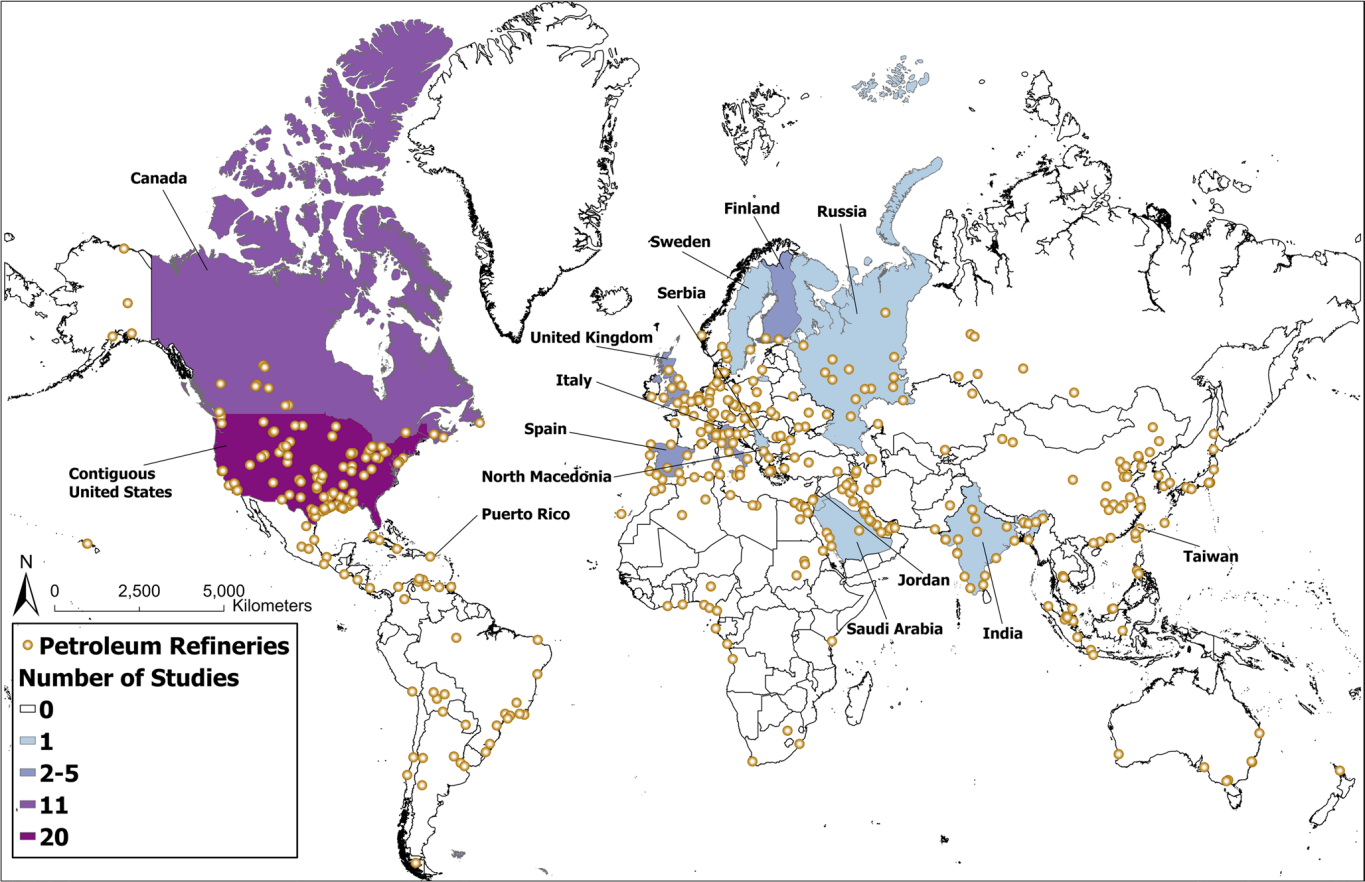
Locations of studies included (labeled) and petroleum refineries (dots). Shading represents the total number of studies conducted in each region. Map lines delineate study areas and do not necessarily depict accepted national boundaries

**Fig. 3:**
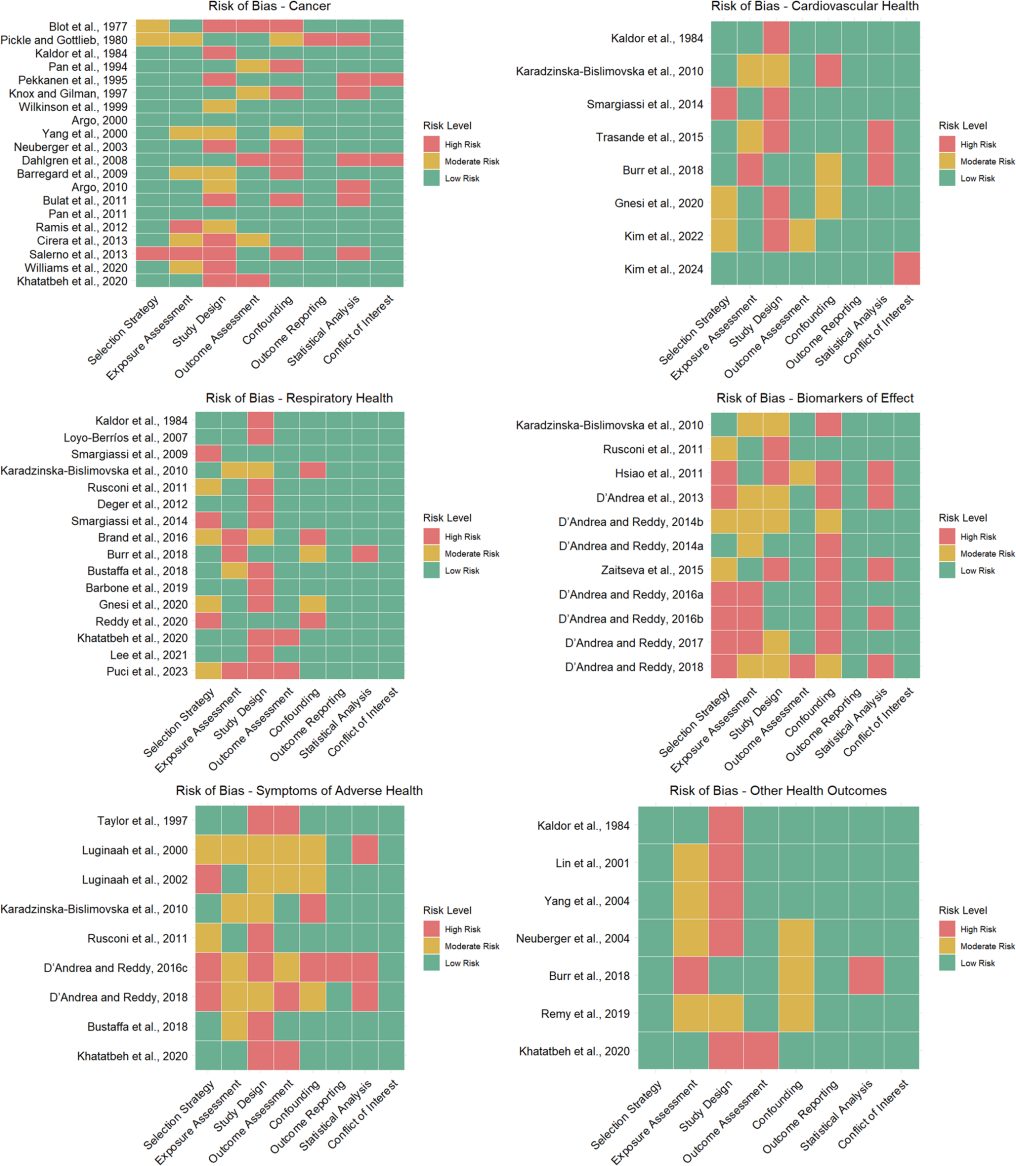
Risk-of-bias heat maps for each study within the six health outcome categories for the following risk-of-bias domains: selection strategy, exposure assessment, study design, outcome assessment, confounding, outcome reporting, statistical analysis, conflict of interest. Studies are listed in chronological order by year of publication. Two authors independently conducted risk-of-bias assessments for each study

**Fig. 4:**
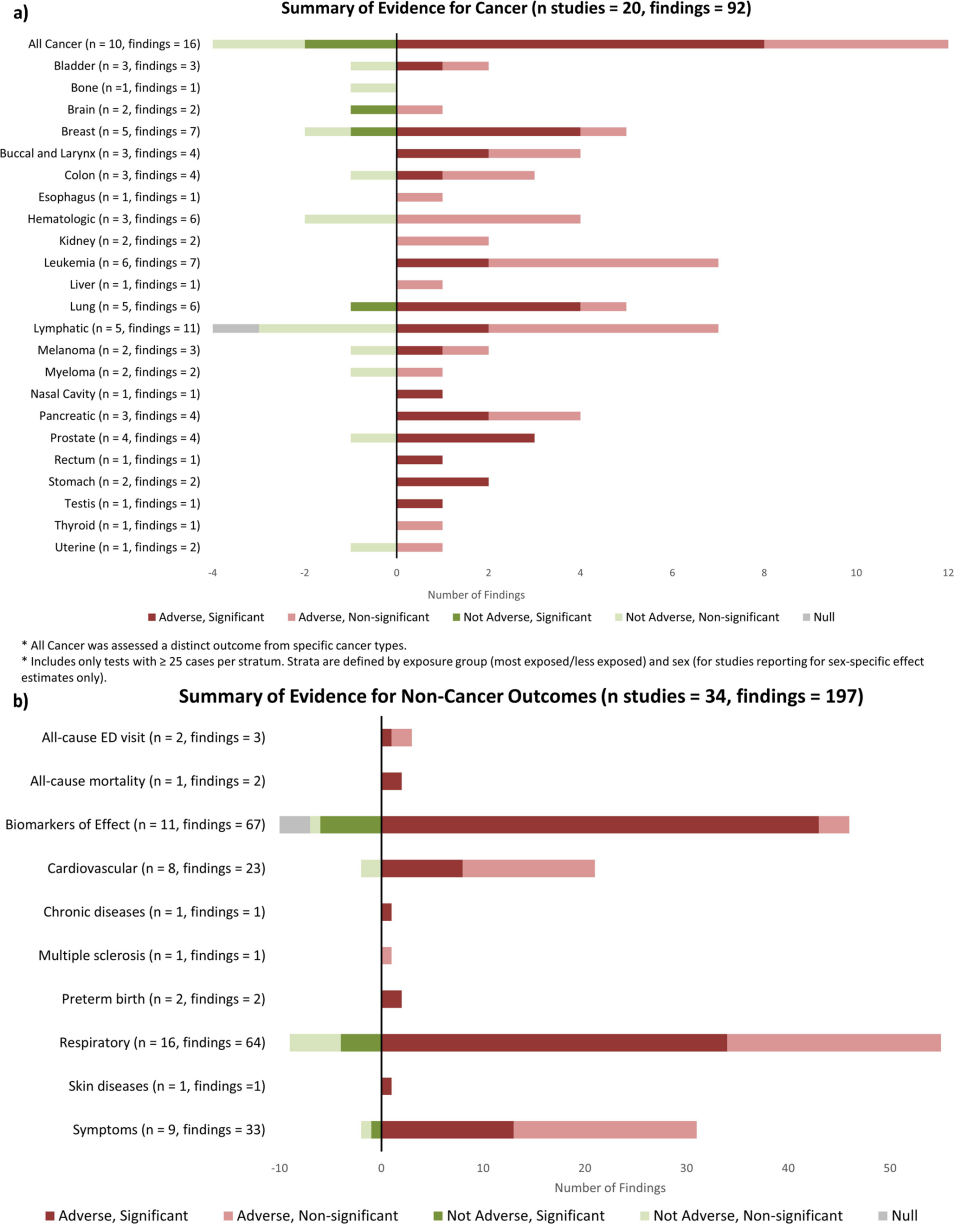
Bar chart summarizing the evidence for all fifty-four studies included in the review for all findings observed in a) twenty studies on cancer and b) thirty-four studies on non-cancer health outcomes. Y-axis comprises the specific cancer type or health outcome, arranged in alphabetical order. X-axis comprises the number of findings reported per stratum. The parentheses in the Y-axis denote the number of studies (n) and the number of findings reported for each type of cancer or health outcome

 The 54 studies included in this review were published between 1977 and 2024 and took place in 15 regions with the majority (n=31) conducted in North America (Fig. 2). Only one study focused on biofuel refineries as opposed to petroleum refineries. Cancer, respiratory effects, biomarkers of effect, acute symptoms, and cardiovascular effects were most studied. Few studies investigated preterm birth, all-cause emergency department visits, all-cause mortality, and other chronic diseases. Risk of bias typically arose from weaknesses in study design (76%), potential confounding (54%), exposure assessment (48%), selection strategy (41%), followed by statistical analysis (30%) and outcome assessment (26%) (Fig. 3). Bias due to selective outcome reporting (4%) and conflict of interest (6%) were uncommon. We reported all findings for each health outcome category across 64 specific health outcomes (Fig. 4).

 Most studies utilized cross-sectional (n=25), followed by ecological (n=15), and case-control designs (n=10), and few were cohort studies (n=4). Exposure was primarily based on proximity where exposed populations were categorized by their location of residence in a specified distance from a refinery (n=39), or exposure was treated as continuous measure of distance from a refinery (n=6). Duration of residence was assessed as a proxy for long-term exposure to refinery emissions (n=7). Some studies estimated ambient air quality using dispersion modeling to predict the spatial distribution of refinery-related pollutants (n=7) or data collected from nearby air monitoring stations (n=3). Few studies assessed associations between individual-level exposure and health outcomes using personal air quality monitors (n=1), urine biomarkers (n=1), or blood samples (n=1).

 In total, 234 of 289 (81%) findings indicate adverse effects among populations near refineries. We observed the most adverse effects among communities living within 5km (59 of 66 findings [89%]) and 10km (31 of 40 findings [78%]) of a refinery compared to those living further away (Table 1). Some evidence suggests adverse effects among residents living up to 20km from a refinery, although only four studies (13 of 14 findings [93%]) considered exposed populations up to this distance. Additionally, we observed adverse associations with closer residential distance to a refinery, estimated continuously per unit decrease in distance (15 of 19 findings [79%]). The evidence suggests health risks are more pronounced closer to the refinery sites which extend beyond fenceline, or directly neighboring, communities.

**Table 1:** Summary of studies and findings for health effects, stratified by the distance between an exposed population and a refinery site up to 20 km. Some studies are counted more than once if they report results for multiple distance categories. Studies that do not specify distance are not included in this table

Distance	No. of Studies	No. of Findings	Results of Findings		
(km)	(*N* = 41)	(*N* = 139)	*Adverse*	*Not Adverse*	*Null*
	N (%)	N (%)	N (%)	N (%)	N (%)
< 5	19 (46)	66 (47)	59 (89)^b^	7 (11)^c^	0 (0)
< 10	11 (27)	40 (29)	31 (78)^d^	8 (20)^e^	1 (2)^f^
≤ 20	4 (10)	14 (10)	13 (93)^g^	(0)	1 (7)^h^
Continuous^a^	7 (17)	19 (14)	15 (79)^i^	4 (21)^j^	0 (0)

^a^ Associations were estimated continuously, per unit decrease in distance of residence to a refinery site

^b^ [26–44]

^c^ [29, 31, 36, 37]

^d^ [35, 37, 40, 45–52]

^e^ [37, 48, 49]

^f^ [37]

^g^ [50, 53–55]

^h^ [50]

^i^ [56–62]

^j^ [59, 62]

### Cancer

 Twenty studies (37%) assessed cancer incidence, prevalence, or mortality for 24 cancer types (Table 2). Several studies reported findings for multiple cancer types within a study population.

**Table 2:** Summary of epidemiological studies of refineries and cancer (*n* = 20 studies)

Reference	Location & Year(s)	Population & Sample Size	Exposure Methods	Findings
Case-Control				
Argo, 2000 [43]	Canada, 1993–1995	Total population 5 km downwind of any refinery in Canada. Cases were people with any cancer and controls were people without cancer. *N* = 2,874 cases, 346 controls	Proximity; categorical: downwind distance of postal code of residence within 2.5 km of refineries.	▲ All-cancer cumulative odds ratio
Argo, 2010 [45]	Canada, 1967–1970	Women (age < 31 years) within 25 km of refineries matched by distance to a refinery and age. Cases were women with pre-menopausal breast cancer cases and controls were women without pre-menopausal breast cancer. *N* = 3,343 cases, 405 controls	Duration: years of residence near a refinery.	▲ Breast cancer odds ratio with longer duration since first exposure to sulfur dioxide and dimethyl sulfate, after a 26-year latency period ǂ
Pan et al., 2011 [28]	Canada, 1994, 1997	Women (age 20–76 years) within 3 km of refineries in 8 provinces matched by age, sex, and province. Cases were women with incident pathologically confirmed breast cancer and controls were women without cancer. *N* = 2,032 cases, 2,215 controls	Proximity; categorical: distance of residence within 3 km of refineries. Duration: years of residence near refineries.	▲ Breast cancer odds ratio among women △ Breast cancer odds ratio among women living near a refinery for more than 1 year
Pickle and Gottlieb, 1980 [38]	US, 1960–1975	Total population within 3 miles of refineries in Louisiana matched by sex, race, age, year of death, and parish of residence. Cases were deaths due to pancreatic cancer and controls were deaths due to other causes. *N* = 82 cases, 71 controls	Proximity; categorical: distance of residence within 1 and 3 miles to refineries. Duration: years of residence within 3 miles a refinery.	▲ Pancreatic cancer mortality odds ratio within 1 mile ▲ Pancreatic cancer mortality odds with longer duration of residence near a refinery ǂ
**Cross-Sectional**				
Khatatbeh et al., 2020 [27]	Jordan, 2018	Adults (≥ 18 years) in Al-Hashimeya, 1 km of a refinery, compared to those in Bal’ma, 12 km to the refinery. Cases were individuals who reported having a family history of cancer. *N* = 486	Proximity; categorical: residence in a town closest to a refinery.	▲ Family history of cancer odds ratio
Knox and Gilman, 1997 [58]	UK, 1953–1980	Children (age 0–15 years) within 10 km of one of 21 refineries, compared to the expected regional and population density adjusted cancer rates. Cases were deaths due to leukemia and all cancer. *N* = 329 cases	Proximity; continuous: distance of postcode of residence to refineries. Exposure effects were assessed per 1 km.	▲ Leukemia or all cancer mortality standardized density ratio within 5 km of refineries*
Pekkanen et al., 1995 [57]	Finland, 1983–1986	Total population within 16 km of one refinery. Cases were incident all cancer diagnoses. *N* = 531 cases	Proximity; categorical: distance of residence to one refinery. Exposure effects were assessed per 4 km.	△ All cancer risk ratio
Ramis et al., 2012 [54]	Spain, 1997–2006	Men and women in towns within 10 km of one of 10 refineries, compared to towns beyond 10 km matched by sociodemographic and industrial characteristics. Cases were non-Hodgkin lymphoma deaths. *N* = 12,229 men and 11,109 women	Proximity; categorical: residence in towns within 10 km of one of 10 refineries.	▲ Non-Hodgkin lymphoma mortality risk ratio for men and women residing near refineries
**Ecological**				
Barregard et al., 2009 [36]	Sweden, 1975–2004	Total population in two parishes within 2 km of a refinery 20 years after the refinery was built, compared to local reference parishes. Cases were incident leukemia and all cancers. *N* = 33 leukemia and 707 all cancer cases	Ambient air quality; dispersion modeling: downwind emissions of benzene to identify exposed parishes.	▲ Leukemia standardized incidence ratio ▽ All cancer standardized incidence ratio
Blot et al., 1977 [63]	US, 1950–1969	White males in 39 counties with petroleum refineries, compared to 117 counties matched by region, population size, and demographic characteristics. Cases were incident cancer deaths. *N* = 39 petroleum-industry and 117 reference counties	Proximity; categorical: residence in a county with a petroleum refinery.	▲ All cancer, stomach, rectum, nasal cavity and sinuses, lung, testis, and melanoma age-adjusted mortality rates △ Buccal and pharynx; esophagus, colon, liver, pancreas, larynx, kidney, bladder, thyroid and endocrine, multiple myeloma, leukemia, and other lymphomas age-adjusted mortality rates ▼ Brain age-adjusted mortality rate ▽ Prostate, bone, and Hodgkin’s age-adjusted mortality rates
Bulat et al., 2011 [65]	Serbia, 2003–2008	Total population in a city with a petroleum refinery, Pančevo, compared to Central Serbia. Cases were incident cancers diagnoses. *N* = 11,647 male and 11,414 female cases	Proximity; categorical: residence in a town with one refinery.	▼ Lung and all cancer incidence rates among men ▼ Breast and all cancer incidence rates among women
Cirera et al., 2013 [59]	Spain, 1996–2007	Total population in 6 cities within 22.5 km of one of 9 refineries. Cases were incident hematological cancer deaths. *N* = 2,574 cases	Proximity; continuous: distance of census tract to one refinery and factored as quintiles. Exposure effects were assessed per quintile decrease in distance.	△ Hematological cancer standardized mortality ratio in 4 cities ▽ Hematological cancer standardized mortality ratio in 2 cities
Dahlgren et al., 2008 [30]	US, 1978–1997	Total population within 1 mile of a retired refinery in Sugar Creek, Missouri which continued to emit benzene, compared to national prevalence rates. Cases were incident Hodgkin lymphoma diagnoses. *N* = 4,854 residents	Proximity; categorical: residence within town near a retired refinery Duration: years exposed to benzene emissions from a retired refinery.	▲Hodgkin’s disease prevalence △ Hodgkin’s disease diagnoses with longer duration exposed to benzene emissions ǂ
Kaldor et al., 1984 [64]	US, 1969–1977	White male and females near 5 refineries in Contra Costa County. Cases were incident cancer diagnoses or deaths. *N* = 15,053 cancer cases; 17,427 deaths	Ambient air quality; dispersion modeling: emissions of SO_2_, HC, and NO_X_ used to group census tracts into 4 areas from least to most exposed.	Men ▲ All cancer standardized mortality ratio ▲ All cancer, buccal and pharynx, stomach, lung, and prostate cancer incidence rates △ Pancreatic, leukemia, and kidney cancer incidence rates ▽ Melanoma, colon, and bladder cancer incidence rates Women ▲ All cancer standardized mortality ratio ▲ Buccal and pharynx cancer incidence rates △ All cancer, colon, lung, melanoma, and cervix uterine cancer incidence rates ▽ Breast and corpus uterine cancer incidence rates
Neuberger et al., 2003 [67]	US, 1986–1999	Total population in a town closest to one refinery, Sugar Creek, compared to a town further away, Independence. Cases were prevalent brain malignant gliomas. *N* = 216 cases	Proximity; categorical: residence in a town closest to one refinery.	△ Brain cancer standardized mortality ratio
Pan et al., 1994 [32]	Taiwan, 1971–1990	Children (age 0–19 years) within 3 km of 3 petroleum and petrochemical complexes in Kaohsiung city, compared to local and national reference rates. Cases were incident cancer deaths. *N* = 132 cases (79 boys, 53 girls)	Proximity; categorical: residence in districts near one refinery.	△ All cancer standardized mortality ratio increased among boys and girls 13 years after complexes expanded △ Leukemia standardized mortality ratio among boys
Salerno et al., 2013 [29]	Italy, 2003–2009	Male and females in Cerano within 3 km of a refinery, compared to those in the district of the Local Health Authority of Novara. Cases were incident cancer diagnoses. *N* = 192 cases	Proximity; categorical: residence in municipality near a refinery. Duration: born in town near a refinery.	▲ Prostate cancer incidence ratios among men ▽ All cancer incidence ratio among men and women ▲ All cancer incidence ratio among residents born in Cerano compared to residents not born in Cerano ǂ
Wilkinson et al., 1999 [37]	UK, 1974–1991	Total population living within 7.5 km of any major refineries in Great Britan, compared to national rates. Cases were incident lymphatic and hematopoietic cancer. *N* = 417 cases within 2 km, 3,827 cases within 7.5 km	Proximity; categorical: residence in postcodes within 2 km and 7.5 km to refineries.	△ All leukemias (acute and chronic lymphoid and myeloid leukemias) and Hodgkin’s lymphoma incidence ratio ▽ Multiple myeloma and non-Hodgkin’s lymphoma incidence ratio
Williams et al., 2020 [55]	US, 2001–2014	Adults (≥ 20 years) within 30 miles to any petroleum refinery in Texas. Cases were incident bladder, prostate, breast, lung, colon, and lymphoma cancer diagnoses. *N* = 283,604 cases	Proximity; categorical: distance of zip code of residence to a refinery and categorized by 10 mile radial buffers areas.	▲ Bladder, lung, prostate, breast, colon, and lymphoma cancer incidence rates* ▲ Distant stage bladder, lung, prostate, breast, colon, and lymphoma cancer incidence rates*
Yang et al., 2000 [66]	Taiwan, 1971–1996	Women in refinery-adjacent municipalities, S. Tso-Ying and Nan-Tzu, compared to national rates. Cases were incident lung cancer deaths. N = not available	Proximity; categorical: residence in a refinery-adjacent municipality.	▲ Lung cancer standardized mortality ratio 30–37 years after refinery operations began

**Direction of Effect**: ▲ Adverse, Significant △ Adverse, Non-Significant ◯ Null ▼ Not Adverse, Significant ▽ Not Adverse, Non-Significant

**Dose-Response**: * Distance to refinery ǂ Duration of exposure or residence near a refinery

### All cancer

 Seven of ten studies (12 of 16 findings [75%]) found a higher risk of all cancer incidence and mortality among adults and children living between 1-16km of petroleum refineries in the US, United Kingdom (UK), Canada, Finland, Jordan, and Taiwan [27, 29, 32, 43, 57, 58, 63, 64] (Table 2). All cancer was assessed as a distinct outcome from specific cancer types. These studies utilized case-control, cross-sectional, and ecological designs. Risk was most severe among those living closest to the refineries [58] and life-long residents of a city with a refinery [29]. Cancer mortality ratios increased among children who lived within 3km of three petrochemical complexes in Taiwan after production expanded 13 years prior, while estimates remained constant in the national and local reference populations [32]. Conversely, three ecological studies in Italy, Serbia, and Sweden found lower all-cancer risk for populations near refineries compared to reference populations. These not adverse findings were affected by substantial limitations with moderate and high risk of bias in study design, statistical analysis, and confounding [29, 36, 65].

### Breast Cancer

 Three of five studies (5 of 7 findings [71%]) found higher risk of breast cancer among women living up to 48km (30 miles) of petroleum refineries in Canada and US compared to those living further away [28, 45, 55] (Table 2). These studies were conducted across statewide or national populations using case-control and ecological designs. Risk was higher among those who were exposed to concentrations of sulfur dioxide and dimethyl sulfate at earlier ages, after a 26-year latency period [45]. Two ecological studies with high risk of bias in study design in Serbia and US observed lower risk of breast cancer among the most exposed populations compared to reference populations [64, 65]. The Serbia study which reported a significant lower risk had high risk of bias in confounding and statistical analysis.

### Leukemia and Other Lymphatic and Hematologic Cancers

 All six studies (6 of 6 findings [100%]) found higher risk of leukemia incidence and mortality among adults and children living within 7.5km of refineries in the UK, Sweden, US, and Taiwan [32, 36, 37, 58, 63, 64] (Table 2). These studies utilized cross-sectional and ecological designs to estimate risk relative to references living further away, less exposed to refinery emissions, or national rates. Notably, 20 years after a refinery was built in Sweden, leukemia risk increased among parishes within 2km to one refinery while remaining constant within reference parishes (Barregard et al., 2009).

 Three of five studies (7 of 9 findings [78%]) reported higher risk of lymphatic cancer, including Hodgkin’s and Non-Hodgkin’s lymphoma, incidence among adults living within 10km of refineries in the US and Spain [30, 54, 55]. These cross-sectional and ecological studies estimated risk relative to reference populations living further away. At a retired refinery which continued to emit benzene in Missouri, US, residents had a seven-fold risk of Hodgkin’s diagnosis, and residents who were chronically exposed to benzene emissions tended to be diagnosed at least 10 years earlier than the average age of diagnosis in the US [30]. Two ecological studies with high risk of bias in study design reported non-significant lower risk of Hodgkin’s, non-Hodgkin's, and other lymphoma incidence and mortality in the US and UK which were largely null [37, 63]. The US study reporting not adverse findings also suffered from high risk of bias in selection strategy, outcome assessment, and confounding due to reporting county-level estimates for several cancers without adjusting for multiple comparisons and covariates other than age [63].

 Two of three (4 of 6 findings [66%]) studies observed higher risk of hematologic cancer, including multiple myeloma, mortality with proximity to refineries in the US and Spain [59, 63]. These ecological studies assessed varying jurisdictional boundaries, from census tracts to counties, however effect estimates were not statistically significant. In contrast, an ecological study with high risk of bias in study design found that postcodes within 2.5 and 7km of refineries showed a non-significant reduction in multiple myeloma risk compared to national UK rates [37].

### Lung Cancer

 Four of five studies (5 of 6 findings [83%]) found higher risk of lung cancer incidence and mortality associated with closer proximity to refineries in the US and Taiwan [55, 63, 64, 66] (Table 2). These ecological studies estimated risk relative to populations living further from refinery sites or are less exposed to refinery-related emissions. Lung cancer risk among women increased in neighboring municipalities 30-37 years after one refinery began operating in Taiwan, while the national estimates remained constant [66]. One study found risk of lung cancer among men was lower in the small city of Pančevo compared to Central Serbia; however, this study’s findings should be interpreted with caution due to the high risk of bias in study design, confounding, and statistical analysis [65].

### Pancreatic Cancer

 All three studies (4 of 4 findings [100%]) found higher pancreatic cancer incidence and mortality risk associated with proximity to refineries in the US [38, 63, 64] (Table 2). Two ecological studies estimated risk relative to reference populations using census tract and county-level units. A case-control study in Louisiana, US conducted on residents within 3 miles (4.8km) of refineries observed that the risk of pancreatic cancer mortality was twice as high among those within 1 mile (1.6km) and cases resided in near refineries longer than controls [38].

### Other Cancers

 Few studies assessed other cancer types among residents up to 20km of refineries in US and Italy (Table 2). Findings from two studies suggest consistently higher risk of incidence and mortality for buccal and larynx (4 of 4 findings [100%]), kidney (2 of 2 findings [100%]), and stomach (2 of 2 findings [100%]) cancers [63, 64]. One study additionally observed higher risk for cancers of the liver, nasal cavity, rectum, testis, esophagus, and thyroid, but non-significant lower risk of bone cancer [63]. Findings were mixed for cancers of the prostate (3 of 4 findings [75%]), melanoma (2 of 3 findings [67%]), bladder (2 of 3 findings [67%]), brain (1 of 2 findings [50%]), and uterus (1 of 2 findings [50%]) [29, 55, 63, 64, 67]. Not adverse findings for these cancer types were reported by two ecological studies which both had high risk of bias in study design [63, 64] and one study also had bias in selection strategy, outcome assessment, and confounding [63].

### Respiratory Effects

 Sixteen studies assessed respiratory health outcomes (Table 3), several of which found that exposure to ambient air pollution near refineries was associated with higher risk of respiratory effects, such as reduced lung function and increased bronchial inflammation [26, 68, 69], healthcare utilization [44, 50, 68, 70], asthma and bronchitis [41, 53], and mortality [64].

**Table 3:** Summary of epidemiological studies of respiratory effects and refineries (*n* = 16 studies)

Reference	Location & Year(s)	Population and Sample Size	Exposure Methods	Results
Cohort				
Barbone et al., 2019 [26]	Italy, 2007	Children (age 8–14 years) attending elementary and middle schools within 1 km of one refinery. *N* = 233 children	Ambient air quality; monitoring: hourly SO_2_ from 3 monitoring stations. Exposure effects were estimated per 10 µg/m3 increase.	▲ Lung function decreases (FEV1 and FEF25-75%) as SO_2_ increases ▲ Bronchial inflammation increases (FeNO) as SO_2_ increases
Burr et al., 2018 [70]	Canada, 1996–2012	Total population in Oakville near one petroleum refinery before and after the refinery closure, compared to nearby populations in the City of Toronto and Greater Toronto. Cases were incident respiratory hospitalizations. *N* = 160,000 residents in Oakville	Proximity; categorical: residence in the city of Oakville before and after the refinery closure.	▲Respiratory hospitalization age and season standardized rates were higher prior to refinery closure and declined after closure
Smargiassi et al., 2014 [68]	Canada, 2009–2010	Children (8–12 years) with asthma, living in a non-smoking home near two refineries. *N* = 72 children	Personal measurements: SO_2_, NO_2_, PM_2.5_, benzene, and PAH via a sampling backpack worn for 10 days. Exposure effects were estimated as the mean change per daily interquartile range increase of each air pollutant.	△ Lung function decreases (FEV1, FVC, and FEF25-75%) as PAHs and benzene increases △ Lung function decreases (FEF25-75%) as NO_2_ and PM_2.5_ increases ▽ Lung function increases (FEV1 and FVC) as NO_2_ and PM_2.5_ increases
**Case-control**				
Brand et al., 2016 [48]	Canada, 2002–2010	Children (age 2–4 years) within 7.5 km of one of 23 refineries in British Colombia and Quebec. Case days were hospital admissions for asthma and bronchiolitis and control days were the same day of the week in the same month. *N* = 2, 868 cases	Ambient air quality: daily downwind NO_2_ and SO_2_ from (1) hourly monitoring stations within 7.5 km of the provinces and (2) dispersion modeling of reported emissions. Exposure effects were estimated per interquartile range of each pollutant for monitored emissions and per 1.50 t/day SO_2_ and 0.40 t/day NO_2_ for reported emissions.	△ Asthma and bronchiolitis hospital admission odds ratio increases as NO_2_ and SO_2_ increases based on ambient monitors ▽ Asthma and bronchiolitis hospital admission odds ratio decreases as NO_2_ and SO_2_ increases based on dispersion modeling of reported emissions
Gnesi et al., 2020 [44]	Italy, 2002–2014	Adults (age 20–64) within 4 km of one refinery matched by age, gender, and municipality. Cases were hospital admissions for respiratory conditions and controls were those not hospitalized over the study period. *N* = 125 cases, 416 controls	Ambient air quality; dispersion modeling: emissions of ambient PM_10_ to estimate exposure at residence.	△ Respiratory-related hospital admissions odds ratio increases with higher exposure to PM_10_
Loyo-Berríos et al., 2007 [56]	Puerto Rico, 1997–2001	Children (age < 17 years) who visited an emergency department, hospital, or physician and matched by gender, age, insurance, and date of asthma event. Cases were incident asthma attacks and controls were asthmatic and non-asthmatic children who did not have an asthma attack on the event day. *N* = 1,382 cases, 6,910 controls	Proximity; continuous: distance of census block of residence to one refinery with wind correction. Exposed effects were estimated per 1 km.	▲ Asthma attacks odds ratio with closer distance*
Smargiassi et al., 2009 [49]	Canada, 1996–2004	Children (2–4 years) within 7.5 km of one of two refineries on the Island of Montreal. Case days were emergency department (ED) visits or hospitalizations for asthma and control days were days of the same day of the week in the same month. *N* = 1,579 ED visits, 263 hospitalizations	Ambient air quality: daily SO_2_ using (1) dispersion modeling from monthly emissions and (2) 2 stationary monitors within 3 km of 2 refineries. Exposure effects were estimated per interquartile range of daily peak levels at post codes of residence.	▲ Asthma-related hospitalizations and ED visit odds ratio increases as SO_2_ increases using dispersion modeling △ Asthma-related hospitalizations and ED visit odds ratio as SO_2_ increases using monitors
**Cross-sectional**				
Bustaffa et al., 2018 [60]	Italy, 2014	Adults (age 17–73 years) in two municipalities adjacent to one refinery frequency matched by gender, age, and municipality. *N* = 100 exposed, 100 references	Proximity; continuous: distance of residence to one refinery. Exposure effects were estimated per 1 km.	▲Severe shortness of breath (dyspnoea) odds ratio with closer distance* △ Chronic bronchitis and bronchial asthma odds ratio with closer distance
Deger et al., 2012 [53]	Canada, 2006	Children (age 6 months-12 years) within 12.8 km of one refinery with and without a medical diagnosis for asthma. Cases of active asthma reported having an asthma diagnosis and active symptoms. Poor asthma control was assessed by symptom-based criteria. *N* = 842 children	Ambient air quality; dispersion modeling: emissions of SO_2_ estimated at location of residence. Exposure effects were estimated per interquartile range increase.	▲ Poor asthma control prevalence ratio increases as SO_2_ increases △ Active asthma prevalence ratio increases as SO_2_ increases
Khatatbeh et al., 2020 [27]	Jordan, 2018	Adults (≥ 18 years) in a town 1 km of one refinery, Al-Hashimeya, compared to a town 12 km from the same refinery. Cases were individuals who reported having a diagnosis or receiving treatment for asthma. *N* = 486	Proximity; categorical: residence in a town closest to one refinery.	▲ Asthma odds ratio
Lee et al., 2021 [50]	US, 2011–2015	Total population (age 1–85 years) within 10 km of 15 biofuel refineries in New York State, compared to residents in 15 control areas not within the radius and matched by census-tract income, age, and race. Cases were respiratory emergency department (ED) visits for emphysema, asthma, chronic bronchitis, and chronic airway obstruction. *N* = 1,285,163 ED visits	Proximity; categorical: distance of residence within 0–5 km and 5–10 km of 15 biorefineries Ambient air quality; dispersion modeling: emissions of NO_2_, SO_2_, and PM_2.5_ estimated up to 20 km for each biorefinery site. Exposure effects were estimated per interquartile range increase of each individual pollutant.	▲Respiratory ED visit rate ratio within 10 km, which was highest within 5 km* ▲Respiratory ED visit rate ratio increases as NO_2_, SO_2_, and PM_2.5_ increases within 5 km △ Respiratory ED visit rate ratio increases as NO_2_ and SO_2_ increases within 10 km ◯ Respiratory ED visit rate ratio increases as PM_2.5_ increases within 10 km
Puci et al., 2023 [41]	Italy, 2016–2019	Adults (20–64 years) within 2–2.5 km of one refinery, compared to the population of Italy. Cases reported diagnosis or treatment for asthma and chronic obstructive pulmonary disease (COPD). *N* = 1,108 adults	Proximity; categorical: residence in two municipalities 2–2.5 km to one refinery.	△ Asthma prevalence △ Asthma-COPD Overlap (ACO) prevalence
Reddy et al., 2020 [46]	India, 2019	Male non-smokers in villages within 5 km of one refinery, compared to controls matched by age and sex living 80 km away from the same refinery. *N* = 200 exposed, 50 references	Proximity; categorical: residence in a village 5 km to one refinery. Duration; categorical: months of residence in the exposed village (6 months, 1 year, 2 years, 3 years).	▲Lung function was lower (FVC, FEV1, PEFR, MMV, FEF25-75%, FEV3, FEV3/FVC, and FEV6) in the exposed village ▲ Lung function was lower (FVC, FEV1, PEFR, MMV, FEF25-75%, FEV3, FEV3/FVC, and FEV6) with longer duration of residence near the refinery ǂ
Rusconi et al., 2011 [69]	Italy, 2006–2007	Children and adolescents (age 6–14 years) in schools near one refinery in Sarroch, compared to those in a nearby rural community in Burcei. Both groups were stratified by those with and without asthma or wheezing. *N* = 275 exposed, 214 reference	Proximity; categorical: attendance in a school near one refinery.	▲ Bronchial inflammation was higher (FeNO) ▲ Lung function was lower (FEV1 and FEF25-75%)
**Ecological**				
Kaldor et al., 1984 [64]	US, 1968–1972	White male and females near 5 refineries in Contra Costa County, California. Cases were deaths due to cardiovascular causes. *N* = 15,053 cancer cases; 17,427 deaths	Ambient air quality; dispersion modeling: emissions of SO_2_, HC, and NO_X_ used to group census tracts into 4 areas from least to most exposed.	△ Respiratory age-adjusted mortality rate in men and women
Karadzinska-Bislimovska et al., 2010 [31]	North Macedonia, 2009	Agricultural workers in a rural community within 300–500 m of one refinery, compared to agricultural workers 21 km to the refinery. *N* = 60 exposed, 59 reference	Proximity; categorical: residence in a community 300–500 m of one refinery.	△ Obstructive ventilatory defects and mixed ventilatory defects were higher ▽ Normal ventilatory capacity, restrictive ventilatory defect and small airways obstruction were lower

**Direction of Effect**: ▲ Adverse, Significant △ Adverse, Non-Significant ◯ Null ▼ Not Adverse, Significant ▽ Not Adverse, Non-Significant

**Dose-Response**: * Distance to refinery ǂ Duration of exposure or residence near a refinery

### Chronic respiratory illnesses

 All four cross-sectional studies (8 of 8 statistical findings [100%]) found consistently higher prevalence of asthma, chronic bronchitis, bronchial asthma, asthma-chronic obstructive pulmonary disease (COPD), and severe shortness of breath among adults and children living near refineries in Italy, Canada, and Jordan compared to reference populations living further away [27, 41, 53, 60] (Table 3). Active asthma and poor asthma control symptoms among children increased with higher exposure to sulfur dioxide (SO_2_) from one refinery in Italy [53]. The prevalence of asthma in the refinery-adjacent population in Italy (12%) was twice as high as the general Italian population, which aligns with the prevalence observed in Jordan (11%) [27, 41].

### Lung function and airway inflammation

 Four of five studies (32 of 39 findings [82%]) observed reduced lung function and increased bronchial inflammation among adults and children living near refineries in Italy, Canada, and India [26, 46, 68, 69] (Table 3). Evidence from two longitudinal cohort studies and two cross-sectional studies were conducted in various populations and provide strong evidence for this association. The studies in Italy were performed by the same investigators using a cross-sectional design to compare lung function between the exposed children and reference municipality, followed by a longitudinal panel study using ambient air quality monitors to estimate changes in lung function with acute exposure to SO_2_ levels [26, 69]. A longitudinal study in Canada assessed individual-level ambient air pollutants and observed lower lung function with exposure to benzene and polycyclic aromatic hydrocarbons (PAHs), but mixed findings with nitrogen dioxide (NO_2_) and fine particulate matter (PM_2.5_) [68]. An ecological study in North Macedonia found mixed associations when comparing an agricultural refinery-adjacent community to reference community further away; however, this study had high risk of bias for confounding due to the lack of control for pesticide exposures and socioeconomic status [31].

### Respiratory hospitalizations and mortality

 Five of five studies found a higher risk of respiratory-related healthcare utilization (5 of 7 findings [71%]) and mortality (2 of 2 findings [100%]) among adults and children living near petroleum refineries in Canada, Puerto Rico, Italy, and the US [44, 48, 56, 64, 70] (Table 3). In addition to ecological designs, some studies utilized quasi-experimental, case-crossover, and case-control approaches. Natural experiments and case-crossover designs in particular can minimize bias that typically arise from traditional observational methods. A natural experiment on the closure of one refinery in Oakville, Canada observed that respiratory hospitalization rates were higher before closure and significantly decreased after closure in the local population, while remaining constant in the reference populations over the same period [70]. A case-crossover study in Canada found higher odds of respiratory hospital admissions among children in provinces downwind of one refinery with higher exposure to NO_2_and SO_2_ based on ambient air monitors, while odds were lower based on dispersion modeling of facility-reported emissions data [48].

 Only one study evaluated health risks in relation to biofuel refineries (Table 3). This cross-sectional study of fifteen biofuel refineries in New York State, US observed that respiratory emergency department visit rates were 264% and 50% higher among residents living within 5km and 5-10km of one of fifteen biofuel refineries than in census tracts matched by income, age, race and region. [50]. The highest rates were among residents living within 5km of biofuel refineries and in descending order for corn, wood, and soybean feedstocks, after adjusting for several place-based socioeconomic and meteorological covariates. PM_2.5_, SO_2_, and NO_2_ based on dispersion modeling were 8-10 times higher within 10km of biofuel refineries compared to air pollutant concentrations beyond 10km.

### Biomarkers of Effect

 Eleven cross-sectional and ecological studies measured biomarkers of effect related to oxidative stress, inflammation, immune, liver, and kidney dysfunction (Table 4). Four cross-sectional studies found elevated biomarkers of immune system dysfunction (5 of 5 findings [100%]), oxidative stress (1 test), systemic inflammation (1 finding), and impaired oxygen transport (1 finding) among adults and children near refineries in Italy, Taiwan, Russia, and India compared to reference areas [31, 69, 71, 72]. Children near refineries in Taiwan and Russia had significantly higher blood levels of lead, toluene, phenol, and formaldehyde than reference groups, suggesting high exposure to toxic contaminants. Children, particularly those with allergies, had higher susceptibility to oxidative stress and immune dysfunction associated with exposure [69, 71, 72].

**Table 4:** Summary of epidemiological studies of biomarkers of effect and refineries (*n* = 11 studies)

Reference	Location & Year(s)	Population and Sample Size	Exposure Methods	Results
Cross-sectional				
D’Andrea et al., 2013 [75]	US, 2010–2012	Children (age < 17 years) and adults (≥ 18 years) within 30mi of a benzene flaring event at one refinery in Texas City, compared to those 30-40mi from the same refinery. *N* = 100 exposed, 100 reference	Proximity; categorical: residence in zip codes within 30mi from one refinery flaring incident.	▲Hematologic marker (WBC and platelet) ▲Hepatic markers (ALP and AST) △ Hepatic marker (ALT) ◯ Hematologic marker (hemoglobin and hematocrit) ▽ Serum creatinine ▼ Blood urea nitrogen
D’Andrea and Reddy, 2014a [73]	US, 2010–2012	Non-smoking adults (age ≥ 18 years) within 30mi of a benzene flaring event at one refinery in Texas City, compared to those 30-50mi from the same refinery. *N* = 1,093 exposed, 329 reference	Proximity; categorical: residence in zip codes within 30mi from one refinery flaring incident.	▲Hematologic markers (WBC, platelet, hemoglobin, hematocrit) ▲Hepatic markers (serum creatinine, blood urea nitrogen, ALP, AST, and ALT)
D’Andrea and Reddy, 2014b [78]	US, 2010–2012	Children (age < 17 years) within 30mi of a benzene flaring event at one refinery in Texas City, compared to those 30-50mi from the same refinery. *N* = 157 exposed, 155 reference	Proximity; categorical: residence in zip codes within 30mi from one refinery flaring incident.	▲ Hematologic marker (platelet) ▲ Hepatic markers (ALP, AST, and ALT) ▼ Hematologic marker (WBC) ◯ Hematologic marker (hemoglobin and hematocrit), blood urea nitrogen, serum creatinine
D’Andrea and Reddy, 2016a [74]	US, 2010–2012	Adults (age ≥ 18 years) within 30mi of a benzene flaring event at one refinery in Texas City, compared to those 30-50mi from the same refinery. *N* = 1,826 exposed, 387 reference	Proximity; categorical: residence in zip codes within 30mi from one refinery flaring incident.	▲ Hematologic markers (WBC and platelet) ▲ Hepatic markers (serum creatinine, ALP, AST, and ALT) ◯ Hematologic marker (hemoglobin and hematocrit), blood urea nitrogen
D’Andrea and Reddy, 2016b [77]	US, 2010	Children (age < 17 years) within 30mi of a benzene flaring event at one refinery in Texas City, compared to those 30-50mi from the same refinery. *N* = 641 exposed, 258 reference	Proximity; categorical: residence in zip codes within 30mi from one refinery flaring incident.	▲ Hematologic marker (platelet) ▲ Hepatic enzymes (ALP, AST, ALT) ◯ Serum creatinine ▼ Hematologic markers (WBC, hemoglobin, hematocrit, blood urea nitrogen)
D’Andrea and Reddy, 2017 [76]	US, 2010–2012	Smoking adults (age ≥ 18 years) within 30mi of a benzene flaring event at one refinery in Texas City, compared to those 30-50mi from the same refinery. *N* = 733 exposed, 58 reference	Proximity; categorical: residence in zip codes within 30mi from one refinery flaring incident.	▲ Hematologic markers (WBC and platelet) ▲ Hepatic markers (ALP, AST, and ALT) ◯ Hematologic markers (hemoglobin and hematocrit), blood urea nitrogen, serum creatinine
D’Andrea and Reddy, 2018 [79]	US, 2010–2012	Adults (age ≥ 18 years) within 30mi of a benzene flaring event at one refinery in Texas City, *N* = 2,162 exposed	Proximity; categorical: residence in zip codes within 30mi from one refinery flaring incident. Exposure effects were categorized by 5 km increments.	▲ Hematologic cancer biomarker (β-2-Microglobulin levels) was highest within 5 and 10 km* ▲ Urinary phenol was highest within 5 and 10 km*
Hsiao et al., 2011 [71]	Taiwan, 2011	Primary school students (grade 5/6) in a community near one refinery in Tao-Yua, compared to children from three urban and rural communities. Children were stratified by those with and without allergies. *N* = 64 exposed, 150 reference	Proximity; categorical: residence in a community near one refinery.	▲ Mean blood lead levels ▲ Humoral immune response was higher (TH2) among children with allergies ▲ Cell-mediated immune response was lower (TH1) among children with allergies
Rusconi et al., 2011 [69]	Italy, 2006–2007	Children and adolescents (age 6–14 years) in schools near one refinery in Sarroch, compared to those in a nearby rural community in Burcei. Both groups were stratified by those with and without asthma or wheezing. *N* = 275 exposed, 214 reference	Proximity; categorical: attendance in a school near one refinery.	▲Oxidative stress (higher MDA-dG adducts)
Zaitseva et al., 2015 [72]	Russia, 2015	Children (age 3–15 years) living near one refining complex, compared to children living in an unexposed territory. *N* = 492 exposed, 165 reference	Proximity; categorical: residence in a community near one refining complex. Personal measurements: blood analysis to estimate of effects of toluene, phenol, and formaldehyde.	▲ Mean blood levels of toluene, phenol, and formaldehyde ▲ Elevated immune response (higher CD19 + lymphocytes) as toluene increases ▲Elevated immune response (higher CD3 + lymphocytes) as formaldehyde increases ▲Immune deficiency (lower IgG) as phenol increases
**Ecological**				
Karadzinska-Bislimovska et al., 2010 [31]	North Macedonia, 2009	Agricultural workers in a rural community within 300–500 m of one refinery, compared to agricultural workers 21 km from the refinery. *N* = 60 exposed, 59 reference	Proximity; categorical: residence in a community 300–500 m from one refinery.	▲ Blood lead level and blood hemoglobin △ Impaired oxygen transport (higher blood carbon monoxide) and systemic inflammation (serum cholinesterase activity)

**Direction of Effect**: ▲ Adverse, Significant △ Adverse, Non-Significant ◯ Null ▼ Not Adverse, Significant ▽ Not Adverse, Non-Significant

**Dose-Response**: * Distance to refinery ǂ Duration of exposure or residence near a refinery

 A series of seven cross-sectional studies in Texas, US evaluated biomarkers of effect using blood and urine samples in a population living near a refinery that released over 500,000 pounds of benzene over 40 days during a flaring event (Table 4). The general adult and non-smoking populations living within 30 miles of the refinery during the flaring event had higher levels of blood biomarkers of systemic inflammation, infection, liver damage, and kidney dysfunction than the population living 30-50 miles from the refinery [73, 74]. These findings were mixed for blood biomarkers in the total population, adults who smoke, and children [75–78]. Liver enzymes were elevated across all subgroups, suggesting implications for liver damage among the exposed population. Biomarkers of hematological cancer and benzene exposure, β-2-microglobulin and urinary phenol, were higher among adults in closest proximity to the flaring event [79]. These studies had moderate and high risk of bias related to selection strategy, exposure assessment, and confounding since the investigators did not adjust for socioeconomic factors.

### Acute Symptoms

 All ten cross-sectional and ecological studies (31 of 33 findings [94%]) observed adverse associations with self-reported health symptoms (Table 5). Children and adults in Italy, Jordan, and North Macedonia reported experiencing more respiratory (cough, wheezing, phlegm) and general (eye, ear, nose, and throat irritation, muscle pain, headache, fatigue) symptoms, as well as neurological, cardiovascular, gastrointestinal, skin, nasal, and osteomuscular issues than those living in further away [27, 31, 60, 69]. After a large-scale benzene flaring event in Texas, adults and children who lived within 5km of the refinery reported more neurological, respiratory, gastrointestinal, and dermatological symptoms compared to those living further away [52, 79].

**Table 5:** Summary of epidemiological studies of health symptoms and refineries (*n* = 9 studies)

Reference	Location & Year(s)	Population and Sample Size	Exposure Methods	Findings
Cross-Sectional				
Bustaffa et al., 2018 [60]	Italy, 2014	Adults (age 17–73 years) in two municipalities adjacent to one refinery frequency matched by gender, age, and municipality. Cases self-reported symptoms. *N* = 100 exposed, 100 reference	Proximity; continuous: distance of residence to one refinery. Exposure effects were estimated per 1 km.	▲ Respiratory allergic symptoms odds ratio △ Cough and sputum not due to common cold odds ratio
D’Andrea and Reddy, 2016c [52]	US, 2010–2012	Children (< 17 years) within 30mi of a benzene flaring event at one refinery in Texas City. Cases self-reported symptoms. *N* = 641	Proximity; categorical: residence in zip codes within 30mi from one refinery, categorized by 5 km increments.	△ Upper respiratory, neurological, diarrhea, cough symptoms were highest within 5 km
D’Andrea and Reddy, 2018 [79]	US, 2010–2012	Adults (≥ 18 years) within 30mi of a benzene flaring event at one refinery in Texas City. Cases self-reported symptoms. *N* = 2,162	Proximity; categorical: residence in zip codes within 30mi from one refinery, categorized by 5 km increments.	△ Neurological, respiratory, cardiac, dermatological, and gastrointestinal were highest within 5 km
Khatatbeh et al., 2020 [27]	Jordan, 2018	Adults (≥ 18 years) in a town 1 km of one refinery, Al-Hashimeya, compared to a town 12 km from the same refinery, in Bal’ma. Cases self-reported symptoms. *N* = 486	Proximity; categorical: residence in a town 1 km from a refinery.	▲ Ear/nose/throat irritation, eye irritation, extreme fatigue, concentration difficulties, memory problems, cough and phlegm symptoms odds ratios
Luginaah et al., 2000 [42]	Canada, 1992, 1997	Adults (≥ 18 years) within 1 km of one petroleum refinery in Oakville, Ontario before and after odor reduction. Cases self-reported symptoms. *N* = 391 (1992), 427 (1997)	Proximity; categorical: distance of residence to one refinery and wind patterns. Exposure effects were categorized by zones with high and low exposure.	△ Respiratory symptoms odds ratio △ General symptoms odds ratio
Luginaah et al., 2002 [62]	Canada, 1992, 1997	Adults (≥ 18 years) and children (< 18 years) within 1 km of one petroleum refinery in Oakville, Ontario before and after odor reduction. Cases self-reported respiratory and general symptoms. *N* = 391 (1992) and 427 (1997)	Proximity; continuous: distance of residence to one refinery and wind patterns.	After refinery odor reduction measures, ▲ Respiratory symptoms odds ratio increased among adults △ Respiratory symptom odds ratio increased among children ▼ General symptoms odds ratio decreased among adults ▽ General symptoms odds ratio decreased among children
Rusconi et al., 2011 [69]	Italy, 2006–2007	Children and adolescents (age 6–14 years) in schools near one refinery in Sarroch, compared to those in a nearby rural community in Burcei. Both groups were stratified by those with and without asthma. *N* = 275 exposed, 214 reference	Proximity; categorical: attendance in a school near one refinery.	▲ Wheezing prevalence among children with and without asthma
Taylor et al., 1997 [61]	Canada, 1992, 1994	Adults (≥ 18 years) and children within 1 km of one petroleum refinery in Oakville, Ontario. Cases self-reported symptoms. *N* = 391	Proximity; continuous: distance of residence to one refinery and wind patterns.	△ Respiratory and general symptoms among adults and children
**Ecological**				
Karadzinska-Bislimovska et al., 2010 [31]	North Macedonia, 2009	Agricultural workers 300–500 m of one refinery, compared to agricultural workers 21 km away. Cases self-reported symptoms. *N* = 60 exposed, 59 reference	Proximity; categorical: residence in community 300–500 m from one refinery.	▲ Muscle pain, headache, fatigue △ Respiratory, cardiovascular, and gastrointestinal symptoms; skin, nasal, and osteomuscular issues

**Direction of Effect**: ▲ Adverse, Significant △ Adverse, Non-Significant ◯ Null ▼ Not Adverse, Significant ▽ Not Adverse, Non-Significant

**Dose-Response**: * Distance to refinery ǂ Duration of exposure or residence near a refinery

 Three consecutive health surveys in Canada from the same research group assessed associations between perceived odors and symptoms of ill health among residents within 1km of one refinery before and after odor reduction measures were implemented [42, 61, 62] (Table 5). Prior to odor reduction measures at the refinery, adults and children living closest to the refinery reported more respiratory and general symptoms than those further away. After odor reduction measures were implemented, general symptoms decreased while respiratory symptoms persisted among those closest to the refinery. These findings suggest that odor control technologies may reduce some health burdens on local communities, while the effect of chronic exposure to refinery emissions and may continue to have lasting health impacts.

## Cardiovascular Effects

### Cardiovascular function

 Three of three studies (15 of 17 findings [88%]) found worse cardiovascular function among children and adults living near refineries (Table 6). Cardiovascular measures, such as blood pressure and pulse rate, were higher on average among adults and children living near refineries than those living further away or less exposed to refinery emissions in Canada, Saudi Arabia, and North Macedonia [31, 39, 49]. In Canada, pulse rate and blood pressure increased among children with higher exposure to PAHs, NO_2_, and benzene, while associations were mixed for PM_2.5_ [68]. Children attending school closest to one refinery in Jordan had higher urine PAH levels, four-fold increased odds of prehypertension, and significantly higher average blood pressure that rose with increasing urine PAH levels [39]. Not adverse findings for pulse rate and systolic blood pressure with exposure to PM_2.5_ were statistically non-significant [68].

### Cardiovascular hospitalizations, morbidity, and mortality

 All four studies found adverse associations for cardiovascular hospitalizations (2 of 2 findings [100%]), mortality (2 of 2 findings [100%]), coronary heart disease (1 finding), and stroke (1 finding) (Table 6). Adults living within 5km of refineries in Italy and the US had a higher risk of cardiovascular-related hospital admissions, mortality, coronary heart disease, and stroke compared to those living further away or less exposed to refinery emissions [35, 40, 44, 64, 70]. Across seven southern states in the US, Hispanic and Black individuals and people with low socioeconomic status were more exposed to petroleum production than White individuals [35]. A natural experiment investigating a refinery closure in Canada found that circulatory hospitalizations were higher prior to closure and decreased after closure within the nearby community, while remaining constant in the reference areas (Burr et al. 2018). Although three of these studies were ecological, the time periods spanned three decades and were conducted in varying regions in the US, suggesting that petroleum refining poses hazards to cardiovascular health that are both widespread and longstanding.

**Table 6:** Summary of epidemiological studies of cardiovascular effects and refineries (*n* = 8 studies)

Reference	Location & Year(s)	Population and Sample Size	Exposure Methods	Findings
Cohort				
Burr et al., 2018 [70]	Canada, 1996–2012	Total population in Oakville near one petroleum refinery before and after the refinery closure, compared to nearby populations in the City of Toronto and Greater Toronto. Cases were incident respiratory hospitalizations. *N* = 160,000 residents in Oakville	Proximity; categorical: residence in the city before and after the refinery closure.	△ Circulatory hospitalization age and season standardized rates were higher prior to refinery closure and declined after closure
Smargiassi et al., 2014 [68]	Canada, 2009–2010	Children (8–12 years) with asthma, living in a non-smoking home near two refineries in an urban industrial area *N* = 72 children	Personal measurements: PAH, NO_2_, benzene, and PM_2.5_ via a sampling backpack worn for 10 days. Exposure effects were categorized as the mean change per daily interquartile range increase of each air pollutant.	*As PAH increased* ▲ Systolic blood pressure decreased △ Pulse rate and diastolic blood pressure increased *As NO*_*2*_ *increased* △ Pulse rate, systolic blood pressure, diastolic blood pressure increased *As Benzene increased* △ Pulse rate, systolic blood pressure, diastolic blood pressure increased *As PM*_*2.5*_ *increased* △ Diastolic blood pressure increased ▽ Pulse rate and systolic blood pressure decreased
**Case-control**				
Gnesi et al., 2020 [44]	Italy, 2002–2014	Adults (age 20–64) within 4 km of one refinery matched by age, gender, and municipality. Cases were hospital admissions for cardiovascular conditions and controls were those not hospitalized over the study period. *N* = 125 cases, 416 controls	Ambient air quality; dispersion modeling: emissions of ambient PM_10_ to estimate exposure at residence	△ Cardiovascular-related hospital admissions odds ratio with higher exposure to PM_10_
**Cross-sectional**				
Trasande et al., 2015 [39]	Saudi Arabia, 2013	Male children (10–14 years) enrolled at and living within 1 km of three schools with varying distances to one refinery (700 m, 9.5 km, and 30.3 km) *N* = 184 boys	Proximity; categorical: enrollment at the school 700 m from one refinery. Personal measurements: urine analysis for effects of PAH.	▲ Prehypertension odds ratio ▲ Systolic blood pressure with higher urine PAH ▲ Diastolic blood pressure with higher urine PAH
**Ecological**				
Kaldor et al., 1984 [64]	US, 1968–1972	White male and females near 5 refineries in Contra Costa County. Cases were deaths due to cardiovascular causes. *N* = 15,053 cancer cases; 17,427 deaths	Ambient air quality; dispersion modeling: emissions of SO_2_, HC, and NO_X_ used to group census tracts into 4 areas from least to most exposed.	▲ Cardiovascular age-standardized mortality rate among men and women
Karadzinska-Bislimovska et al., 2010 [31]	North Macedonia, 2009	Agricultural workers living 300–500 m from one refinery compared to agricultural workers 21 km from the refinery *N* = 119 participants	Proximity; categorical: residence in community 300–500 m from one refinery.	△ Systolic blood pressure average △ Diastolic blood pressure average
Kim et al., 2022 [40]	US, 2018	Adults (≥ 18 years) in 7 states in the US South residing in census tracts within 2.5 and 5 km of one of 59 refineries, compared to census tracts 5–11 km from one refinery. Cases were those who reported having a medically diagnosed stroke. *N* = 1,171 census tracts	Proximity; categorical: wind- and distance- weighted average of petroleum production in census tracts within 2.5 km and 2.5-5 km of one refinery. Exposure effects were categorized per-standard deviation increase of petroleum production (PPR).	▲ Stroke prevalence as exposure to PPR increases
Kim et al., 2024 [35]	US, 2018	Adults (≥ 18 years) in 7 states in the US South residing in census tracts within 2.5 and 5 km of one of 59 refineries, compared to census tracts 5–11 km from one refinery. Cases reported a diagnosis for coronary heart disease. *N* = 1,171 census tracts	Proximity; categorical: wind and distance weighted average of petroleum production in census tracts within 2.5 km and 5 km of one refinery. Exposure effects were categorized per-standard deviation increase of petroleum production (PPR).	▲ Coronary heart disease prevalence as exposure to PPR increases*

**Direction of Effect**: ▲ Adverse, Significant △ Adverse, Non-Significant ◯ Null ▼ Not Adverse, Significant ▽ Not Adverse, Non-Significant

**Dose-Response**: * Distance to refinery ǂ Duration of exposure or residence near a refinery

### Other Adverse Health Outcomes

 Two case-control studies (2 of 2 findings [100%]) found adverse findings for preterm birth, both of which were based in Taiwan and conducted by the same research group (Table 7). Both studies found higher odds of preterm birth, defined as delivery prior to 37 completed weeks, among mothers living within 3km of one refinery compared to the rest of Taiwan while controlling for individual-level and seasonal covariates [33, 34]. The first study included mothers near one petroleum refinery, and the later study included mothers near one of three refineries.

**Table 7:** Summary of epidemiological studies of other adverse health outcomes and refineries (*n* = 7 studies)

Reference	Location & Year(s)	Population and Sample Size	Exposure Methods	Finding
Cohort				
Burr et al., 2018 [70]	Canada, 1996–2012	Total population in Oakville near one petroleum refinery before and after the refinery closure, compared to nearby populations in the City of Toronto and Greater Toronto. Cases were incident respiratory hospitalizations. *N* = 160,000 residents in Oakville	Proximity; categorical: residence in the city of Oakville before and after the refinery closure.	△ All-cause hospitalization age and season standardized rates were higher prior to refinery closure and declined after closure
Remy et al., 2019 [47]	US, 2007, 2012	Residents in Contra Costa County four weeks before and after refinery fires in 2007 and 2012. Cases were incident all-cause emergency department (ED) visits. *N* = 105,020 ED visits	Proximity; categorical: distance of zip-code of residence to one refinery and categorized as 0-4mi, 4-10mi, and > 10mi. Temporal period was categorized as 4 weeks before and after the fires.	▲ All-cause ED visit rates were higher after the refinery fire than before in zip-codes within 10 mi, which was highest within 4mi*
**Case-Control**				
Lin et al., 2001 [34]	Taiwan 1993–1996	First-parity singleton births within 3 km of one refinery, compared to the rest of Taiwan. Cases were preterm births and controls were non-preterm births. *N* = 2,027 exposed (62 cases); 49,673 controls (1131 cases)	Proximity; categorical: distance of residence within 3 km of one refinery.	▲ Preterm birth odds ratio
Yang et al., 2004 [33]	Taiwan, 1994–1997	First-parity singleton births within 3 km of one of three refineries, compared to the rest of Taiwan. Cases were preterm births and controls were non-preterm. *N* = 7,095 exposed (349 cases); 50,388 controls (2213 cases)	Proximity; categorical: distance of borough of residence within 3 km of one of three refineries.	▲ Preterm birth odds ratio
**Cross-Sectional**				
Khatatbeh et al., 2020 [27]	Jordan, 2018	Jordanian citizens (≥ 18 years) in a town 1 km from one refinery, Al-Hashimeya, compared to a town 12 km from the same refinery, in Bal’ma. Cases self-reported having skin or chronic diseases. *N* = 486	Proximity; categorical: residence in a town 1 km from one refinery.	▲ Skin diseases odds ratio ▲ Chronic diseases odds ratio
Neuberger et al., 2004 [80]	US, 1998–2001	Residents in a town closest to one refinery, Sugar Creek, compared to a town further away, Independence. Cases were multiple sclerosis diagnoses. *N* = 142 cases	Proximity; categorical: residence in a town closest to one refinery.	△ Multiple sclerosis prevalence
**Ecological**				
Kaldor et al., 1984 [64]	US, 1968–1972	White male and females near 5 refineries in Contra Costa County. Cases were incident deaths for any cause. *N* = 17,427 deaths	Ambient air quality; dispersion modeling: emissions of SO_2_, HC, and NO_X_ used to group census tracts into 4 areas from least to most exposed.	▲ All-cause age-standardized mortality ratio among men and women

**Direction of Effect**: ▲ Adverse, Significant △ Adverse, Non-Significant ◯ Null ▼ Not Adverse, Significant ▽ Not Adverse, Non-Significant

**Dose-Response**: * Distance to refinery ǂ Duration of exposure or residence near a refinery

 Two studies (3 of 3 findings [100%]) found higher risk of other chronic conditions in the US and Jordan (Table 7). These cross-sectional studies found that adults living at the fenceline of refineries had higher risk of multiple sclerosis, a chronic autoimmune disease, as well as skin diseases and chronic diseases compared to those living further away [27, 80].

 Three of three studies found adverse findings for all-cause healthcare utilization (2 of 2 findings [100%]) and mortality (1 finding) (Table7). Two natural experiments and one ecological study in the US and Canada observed that adults living closest to refineries had higher risk of all-cause emergency department visits, hospitalizations, and mortality compared to those living further away or less exposed [47, 64, 70]. These natural experiments offer contrasting yet compelling evidence on how local healthcare systems are affected by refinery emissions. Emergency department (ED) visits significantly increased for four weeks after two fires at one refinery in the US [47], and hospitalizations were higher prior to closure and decreased in a nearby city after a refinery retired in Canada which remained constant in reference areas [70].

## Discussion

 Among the 54 observational studies included in this review, 64 specific health outcomes were assessed across 47 years of evidence that were primarily conducted on petroleum refineries in the US and Canada. We catalogued the literature into a systematic evidence map by health outcome category, exposure methods, type of refinery, study design, and population characteristics. Additionally, we synthesized and evaluated the methodological quality of the epidemiological literature regarding petroleum and biofuel refineries and community health. Most findings indicate adverse health effects associated with residential proximity to petroleum refineries. Evidence of adverse effects were consistent for leukemia, pancreatic, buccal and larynx, and stomach, and kidney cancers. Associations for all cancer types combined and lung, breast, lymphatic, hematologic, and prostate cancers were primarily adverse. Limited findings for other cancer types, such as brain cancer and melanoma, were mixed. Further, evidence indicates that cardiorespiratory diseases (asthma, bronchitis, heart disease, stroke), respiratory impairment, mortality, excess healthcare utilization, and biomarkers of systemic inflammation, immunodeficiency, liver, and kidney dysfunction are higher among adults and children living near petroleum refineries. We found some evidence of adverse effects for preterm birth, neurological symptoms, oxidative stress, dermatological and autoimmune diseases, though only a few studies assessed these outcomes. Adverse effects were most pronounced among residents closest to or living near refineries for longer durations. 

 Study quality has improved over time with advancements in designs (e.g. natural experiments and case-crossover approaches) and exposure metrics (e.g. pollution dispersion modeling and individual-level monitoring) that reduce the potential for exposure misclassification bias. However, most studies to date have been ecological or cross-sectional, making it difficult to establish causality. Acute respiratory, immune, and liver effects suggest that long-term residence near refineries may contribute to chronic respiratory and autoimmune diseases. Two prospective studies indicated reduced lung function and increased bronchial inflammation among children when exposed to higher levels of PAHs, SO_2_, and benzene from petroleum refineries [26, 68]. Two natural experiments emphasize how, in both fires at and the closure of refineries, petroleum production near communities significantly burdens healthcare demand and underlying population health [47, 70].

 Dose-response relationships by distance (8 studies) and duration of residence (5 studies) near petroleum and biofuel refineries demonstrate strong evidence of adverse health impacts. Adults and children living closest to a refinery, particularly within 5km downwind, had the highest risks of cancer mortality, stroke, coronary heart disease, respiratory emergency department visits, asthma attacks, and severe shortness of breath than populations further away [35, 40, 50, 56, 58, 60]. Risks declined with distance but remained elevated up to 10km for some outcomes. Associations with distance were stronger when weighted by production capacity [35, 40]. Large-scale toxic emission releases were linked to elevated all-cause emergency department visit rates and biomarkers of cancer and benzene exposure, with the highest levels observed among residents within 5km from the refinery and effects remaining elevated, though attenuated, up to 20km away [47, 79]. Residents living near refineries for longer periods had higher risks of cancer incidence, cancer mortality, and impaired lung function [29, 30, 38, 45, 46]. These health disparities vary by factors such as proximity to refineries, duration of exposure, wind patterns, and the scale of production or emissions. Therefore, the exact distance at which health impacts decline remains uncertain, particularly due varied definitions of exposed and reference populations in the existing literature. Our review supports further dose-response assessments and future meta-analyses to reach conclusions.

 Only one study to date assessed the health impacts of biofuel refineries. Elevated levels of PM_2.5_, SO_2_, and NO_2_ and excess respiratory-related emergency department visits were associated with biofuel refineries across various feedstock types [50]. These findings align with peer-reviewed and gray literature indicating that biofuel refineries, particularly those using corn and sugarcane feedstocks, emit greenhouse gasses and hazardous air pollutants at levels equivalent or greater in magnitude than those from petroleum refineries [8, 81, 82]. Recent low carbon fuel standard policies adopted by several jurisdictions, such as in Oregon and California in the US and British Columbia in Canada, incentivize conversions of petroleum-based refineries to produce biofuel [83]. However, transitions from petroleum to biofuel refineries may perpetuate – rather than mitigate – air quality issues that threaten the health of local communities. Strengthening research and monitoring of biofuel refineries is critical to better understand their health implications and to inform decisions regarding broad-scale transitions from petroleum to biofuel refining.

 Studies on susceptible populations and changes in refinery emissions provide meaningful evidence of refinery-related health impacts and the need for regulatory oversight. Children living or attending school near refineries were consistently found to have higher risks of cancer, acute and chronic respiratory conditions, and immune system dysfunction [26, 56, 58, 69, 71]. Sudden toxic emission releases from fire and flaring events inflict disproportionate health risks and strain local healthcare systems in nearby communities [47, 79]. Facility closures and technological controls at refineries have been shown to reduce health risks, highlighting the need for regulations aimed at controlling emissions and protecting vulnerable populations [62, 70]. Yet, retired refineries leave behind a legacy of sacrifice zones and enduring harm without extensive remediation of land and water contamination [30]. Moreover, penalizing facilities for years of improperly managed or underreported pollution, as in the case of an $82 million dollar settlement for two decades of unreported toxic emissions at a refinery in California, US, does little to mitigate the legacy of prior community health impacts [84]. Rather, it is paramount that regulatory standards including air quality control measures, emission reporting standards, and cleanup in the wake of refinery closures are proactively enforced with community partnership in regulatory oversight [85].

## Limitations

 The most common measure of exposure to refineries was residential proximity. Studies that assessed exposure based on residence in a large geographical region, such as counties, were more likely to report inconclusive findings than studies that used smaller area-level units, such as zip-codes, or calculated residential proximity using point-level residential addresses. Exposure misclassification and residual confounding were common risks of bias since adjustments were often overlooked for socioeconomic status, residential mobility, wind direction, and background air pollution. Some studies estimated exposure based ambient and personal air quality monitoring as well as dispersion modelling of industry-reported refinery emissions. While this represents an improvement over crude proximity-based measures, these approaches do not account for other types and routes of exposure (e.g. pollutant emissions to water, noise, light pollution, or psychosocial stress associated with perceived health threats). Refinery emissions are often underreported, which could result in underestimates of exposure [86, 87].

 These studies assessed health outcomes varying from administrative health records, self-reported symptoms, to biomarker measurements. Many studies relied on secondary data from administrative healthcare records, which may underestimate the full health burden since only severe cases are included or lead to exposure misclassification when used without residential history information. Additionally, studies that assessed multiple cancer types simultaneously or did not account for cancer latency periods were more prone to bias due to multiple comparisons and inadequate length of follow-up. Self-reported symptoms are to be interpreted with caution if studies do not control for reporting bias.

 Although we aimed to conduct a thorough and comprehensive review, we acknowledge some limitations. First, we cannot guarantee that every relevant study was identified although we performed backwards and forwards citation searches to mitigate this issue. By restricting to peer-reviewed literature, we may have excluded insights from the gray literature. Although we did not specifically exclude non-English studies in our search, we did not identify publications in languages other than English. Therefore, regions with active refinery operations where English is not the primary language (e.g. South America) may be underrepresented in our analysis. Second, our synthesis is descriptive as our aim was to summarize the breadth of potential health effects. A meta-analysis was not feasible due to substantial heterogeneity in study designs, populations, exposure assessment methods, and measures of association across health outcomes [88]. Third, we did not assess certainty in the body of evidence for each health outcome. We opted to retain the scores for each risk-of-bias domain for each study to account for each domain’s distinct importance, rather than scoring the overall quality of each study. Certainty regarding the strength of the evidence of each health outcome includes summarizing the consistency of effects across findings from multiple studies (Fig. 4).

### Research and Policy Recommendations

 Future research can improve methods to advance our understanding of refinery-related health impacts, including:

1. Longitudinal and quasi-experimental designs that assess temporality and better establish causality
2. More spatially and temporally resolved exposures and modeling, as well as undertaking personal exposure measurements that better capture individual-level exposures. When defining exposure as distance to a refinery, researchers should report effect estimates stratified by distance (for example, per 1 or 2 km) to enable future meta-analyses and dose-response analyses. When using units such as cities or counties, researchers should report the range of distance to the nearest refinery to improve interpretability.
3. Statistical testing of outcome-specific hypotheses defined *a priori* to reduce bias related to multiple comparisons and small stratum sizes.
4. Better control for unmeasured confounding
5. Assessment of effect measure modification based on individual and placed-based factors

Susceptibility to the adverse effects associated with emissions from refineries may be related to age, socioeconomic status, co-morbidities, and pregnancy, among other factors. Co-exposures to social stressors in fenceline communities related to poor housing quality, food insecurity, and unemployment may amplify the health effects of refinery-related environmental exposures; yet no studies to date have assessed effect measure modification by factors other than age, sex, and asthmatic or allergic status. There is a pressing need for further research on reproductive and neurological outcomes, as well as the cumulative effects of chronic exposure to pollutant mixtures and compounding effects of exposures to social stressors. Research should focus on identifying the populations disproportionately impacted by the refinery industry. Proximity to refineries is rooted in systematic inequities as economically disadvantaged and racially minoritized households often live closer to refineries [5, 35]. Community-based participatory research approaches that engage impacted community members to co-create knowledge could help identify novel hypotheses, improve study rigor, and extend the reach of findings [89, 90]. Bridging these knowledge gaps can inform and support future policy decisions that better protect community health.

 The evidence in this review points to the need for policy and regulatory frameworks to prioritize equity when addressing new and persistent challenges posed by existing, retiring, and new petroleum and biofuel refineries. Community input in decisions related to refinery siting, air quality permitting, and the enforcement of buffer zones between refineries and populations is necessary to reduce exposure near homes, schools, workplaces, and healthcare facilities. Most important, community partnership in planning energy transitions provides a crucial opportunity to change the course on longstanding environmental injustice and health disparities. Local, regional, and international policies are crucial to facilitate the shift from reliance on polluting energy inputs to the adoption of cleaner technologies, while ensuring that potential unintended consequences are avoided in the pursuit of sustainable development strategies that advance climate change, public health, and environmental justice goals.

## Conclusion

 To our knowledge, this is the first systematic evidence map and scoping review of the range of health impacts among communities living near refineries. Evidence spanning fifteen regions worldwide, over four decades, and various study designs overwhelmingly suggests that petroleum and biofuel refineries emit toxic air pollutants which disproportionately harm the health of nearby adults and children. It is evident that refinery communities face significantly higher risks of cancers (leukemia, pancreatic, and buccal and larynx), mortality, asthma, bronchitis, respiratory impairment, worse cardiovascular function, excess healthcare utilization, and acute symptoms. Limited evidence also suggests higher risk of preterm birth, immunodeficiency, systemic inflammation, neurological effects, liver and kidney dysfunction, though further investigations are needed for these outcomes. Children, individuals with pre-existing conditions, and those living closest to refineries are particularly vulnerable to pollutants emitted by these facilities. While some findings were not consistent across all studies, potentially owing to the high variation in methodological quality and regional contexts, the health burden imposed by refineries is widespread and longstanding. Our risk-of-bias assessments support future research with improvements in design, exposure methods, and control for unmeasured confounding to strengthen the evidence base. Future work is urgently needed to fill data gaps identified in this review regarding the consequences of biofuel refineries, the impacts on reproductive and neurological outcomes, and the compounding effects of socioeconomic vulnerability. Our review contributes an evidence base and new knowledge to support science-based decision-making concerning health disparities in overburdened communities and a just energy transition.

## Supplementary Information

Below is the link to the electronic supplementary material.

Supplementary Material 1 (DOCX 23.3 KB)

## Data Availability

The systematic evidence map is available via Tableau:https://public.tableau.com/app/profile/refineryreview/viz/Refinery-SEM/EvidenceMap.
