# Supplementary material for: Health Effects of Living Near Petroleum and Biofuel Refineries: A Systematic Evidence Map and Scoping Review
Source: Curr Environ Health Rep. 2026 Mar 14;13(1):11. doi: 10.1007/s40572-026-00530-0 (PMC12988906; doi:10.1007/s40572-026-00530-0)
Supplement: Supplementary file 1 — Supplementary Material 1 (DOCX 23.3 KB) [file 40572_2026_530_MOESM1_ESM.docx]

**Supplementary Materials for**

**Title: Health Effects of Living Near Petroleum and Biofuel Refineries:** **A Systematic Evidence Map and Scoping Review**

**Authors and Affiliations:**

Marinelle Villanueva^a^ , Qi Meng^a^, Jenni A. Shearston^b,c^, Rachel Morello-Frosch^c^, Lara J. Cushing^a^

^a^Department of Environmental Health Sciences, Fielding School of Public Health, University of California, Los Angeles, Los Angeles, CA, USA

^b^Department of Integrative Physiology, University of Colorado Boulder, Boulder, CO, USA

^c^Department of Environmental Science, Policy, and Management, and School of Public Health, University of California, Berkeley, Berkeley, CA, USA

**Correspondence:** Lara J. Cushing, Ph.D., M.P.H., <lcushing@ucla.edu>

**This file includes:**

Glossary

Table S1. Database search strategies

Table S2. Critical appraisal checklist for risk of bias assessments

**Glossary**

Dispersion modeling: a computational method used to estimate how air pollutants spread and dilute in the atmosphere from emission sources based on factors such as wind, temperature, and topography

Feedstock: raw or processed material used as input in a petroleum or biofuel refinery to liquid fuels. In petroleum refineries, this primarily includes crude oils. In biofuel refineries, this can include vegetable oils (e.g. soybean, canola, and palm oil), animal fats, recycled cooking oils, and biomass such as algae, crop residues, and dedicated crops

NO_2_: nitrogen dioxide

PAH: polycyclic aromatic hydrocarbons

PM_2.5_: particulate matter less than 2.5 μm in aerodynamic diameter

PM_10_: particulate matter less than 10 μm in aerodynamic diameter

SO_2_: sulfur dioxide

***Supplemental Table S1*** Search strategy utilized to identify literature in PubMed, Embase, and Web of Science databases. The database search was initiated in February 2024

| **Database** | **Keyword** | **Search Terms** |
| --- | --- | --- |
| **PubMed** | Refining  AND  Health  AND  Proximity | "Oil and Gas Industry"[Mesh] OR "oil refin*" OR "petroleum refin*" OR "Natural-gas processing" OR "gas processing" OR ("downstream" AND (Oil OR Gas)) OR (("Petroleum"[Mesh] OR "Natural Gas"[Mesh]) AND (Refining OR Refinery)) OR (("Biofuels"[Mesh] OR Biorefiner* OR Biogas OR Biodiesel) AND (Refining OR Refinery))  AND  ("Environmental Exposure"[Mesh] OR "Public Health"[Mesh] OR “Community Health” OR "Environmental Health"[Mesh] OR “Human Exposure*” OR “Health Outcome*” OR “Health Effect*” OR "Respiration Disorders"[Mesh] OR “respiratory illness” OR "Hospitalization"[Mesh] OR "Emergency Room Visits"[Mesh] OR “Emergency Room Visit*” OR "Neoplasms"[Mesh] OR Cancer OR Tumor* OR "Alzheimer Disease"[Mesh] OR "Congenital Abnormalities"[Mesh] OR "Air Pollution"[Mesh] OR “Air Quality” OR "Particulate Matter"[Mesh] OR "Volatile Organic Compounds"[Mesh] OR VOC OR "Benzene"[Mesh] OR "Mortality"[Mesh] OR “residential exposure”)  AND  (“Residential” OR “Near*” OR “Proximity”) |
| **Embase** | Refining  AND  Health  AND  Proximity | ('oil industry'/exp OR 'oil refin*' OR 'petroleum refin*' OR 'natural-gas processing' OR 'gas processing' OR (downstream AND (oil OR 'oil'/exp OR 'gas'/exp OR gas)) OR (('petroleum'/exp OR 'natural gas'/exp) AND (refining OR refinery)) OR (('biofuel'/exp OR biorefiner* OR biogas OR biodiesel) AND (refining OR refinery)))  AND  ('environmental exposure'/exp OR 'public health'/exp OR 'community health' OR 'environmental health'/exp OR 'human exposure*' OR 'health outcome*' OR 'health effect*' OR 'breathing disorder'/exp OR 'respiratory tract disease'/exp OR 'respiratory illness' OR 'hospitalization'/exp OR 'breathing disorder' OR 'malignant neoplasm'/exp OR 'cancer' OR 'tumor' OR 'alzheimer disease'/exp OR 'congenital disorder'/exp OR 'air pollution'/exp OR 'air quality'/exp OR 'particulate matter'/exp OR 'volatile organic compound'/exp OR voc OR 'benzene'/exp OR 'mortality'/exp  AND  ('residential' OR ‘near*’ OR ‘proximity’) |
| **Web of Science** | Refining  AND  Health  AND  Proximity | ("Oil and Gas" OR "oil refin*" OR "petroleum refin*" OR "Natural-gas processing" OR "gas processing" OR ("downstream" AND (Oil OR Gas)) OR (("Petroleum" OR "Natural Gas") AND (Refining OR Refinery)) OR (("Biofuels" OR Biorefiner* OR Biogas OR Biodiesel) AND (Refining OR Refinery)))  AND  ("Environmental Exposure" OR "Public Health" OR “Community Health” OR "Environmental Health" OR “Human Exposure*” OR “Health Outcome*” OR “Health Effect*” OR "Respiration Disorder*" OR "Respiratory Disorders" OR “respiratory illness” OR "Hospitalization" OR "Emergency Room Visit*" OR "Neoplasm*" OR Cancer OR Tumor* OR "Alzheimer’s Disease" OR "Congenital Abnormalit*" OR "Air Pollution" OR “Air Quality” OR "Particulate Matter" OR "Volatile Organic Compound*" OR VOC OR "Benzene" OR "Mortality" OR “residential exposure”)  AND  (“Residential” OR “Near*” OR “Proximity”) |

***Supplemental Table S2*** Evaluation checklist to critically appraise risk of bias. Two reviewers used these criteria to independently assess individual study quality

| **Critical Appraisal Checklist** |
| --- |
| *Selection Strategy*   1. Were the criteria for inclusion in the sample clearly defined and the study subjects and the setting described in detail? (YES = 1, NO = 0) 2. Does the study population reasonably reflect the target population, or is there risk of selection bias due to the recruitment strategy or loss to follow up? (YES = 1, NO = 0) |
| *Exposure Assessment*   1. Was the exposure measured in a standard (same for all groups) way? (YES = 1, NO = 0) 2. Was the exposure measured in a valid and reliable way? (YES = 1, NO = 0) |
| *Study Design*   1. Did the health outcome occur after the exposure? Was temporality addressed appropriately? (YES = 1, NO = 0) 2. Was the exposure period of interest meaningful for the health outcome? For cohort studies, was the length of follow up adequate? (YES = 1, NO = 0) |
| *Outcome Assessment*   1. Was the outcome measured in a standard (same for all groups) way? (YES = 1, NO = 0) 2. Was the outcome measured in a valid and reliable way? (YES = 1, NO = 0) |
| *Selective Outcome Reporting*   1. Were all outcomes specified in the methods reported? (YES = 1, NO = 0) |
| *Confounding*   1. Were confounding factors identified? (YES = 1, NO = 0) 2. Were strategies to deal with confounding factors stated and used (e.g., matching, adjustment, comparability of groups)? (YES = 1, NO = 0) |
| *Statistical Analysis*   1. Was the statistical analysis clearly described and appropriate? (YES = 1, NO = 0) |
| *Conflict of Interest*   1. Does the conflict-of-interest statement or author affiliations indicate there is no financial interest in the outcome of the study by any individual or entity? (YES = 1, NO = 0) |
